# Phylogeny and gene expression of the complete *NITRATE TRANSPORTER 1/PEPTIDE TRANSPORTER FAMILY* in *Triticum aestivum*

**DOI:** 10.1093/jxb/eraa210

**Published:** 2020-05-28

**Authors:** Huadun Wang, Yongfang Wan, Peter Buchner, Robert King, Hongxiang Ma, Malcolm J Hawkesford

**Affiliations:** 1 Institute of Food Crops, Provincial Key Laboratory of Agrobiology, Jiangsu Academy of Agricultural Sciences, Nanjing, China; 2 Rothamsted Research, West Common, Harpenden, UK; 3 Nanjing Agricultural University, China

**Keywords:** Gene expression, nitrate, nitrogen, *NPF* gene, phylogeny, wheat (*Triticum aestivum*)

## Abstract

*NPF* genes encode membrane transporters involved in the transport of a large variety of substrates including nitrate and peptides. The *NPF* gene family has been described for many plants, but the whole *NPF* gene family for wheat has not been completely identified. The release of the wheat reference genome has enabled the identification of the entire wheat *NPF* gene family. A systematic analysis of the whole wheat *NPF* gene family was performed, including responses of specific gene expression to development and nitrogen supply. A total of 331 *NPF* genes (113 homoeologous groups) have been identified in wheat. The chromosomal location of the *NPF* genes is unevenly distributed, with predominant occurrence in the long arms of the chromosomes. The phylogenetic analysis indicated that wheat *NPF* genes are closely clustered with Arabidopsis, *Brachypodium*, and rice orthologues, and subdivided into eight subfamilies. The expression profiles of wheat *NPF* genes were examined using RNA-seq data, and a subset of 44 *NPF* genes (homoeologous groups) with contrasting expression responses to nitrogen and/or development in different tissues were identified. The systematic identification of gene composition, chromosomal locations, evolutionary relationships, and expression profiles contributes to a better understanding of the roles of the wheat *NPF* genes and lays the foundation for further functional analysis in wheat.

## Teaser

This first systematic characterization of the whole wheat *NPF* gene family contributes to a better understanding of this large gene family in wheat and lays the foundation for further functional analysis.

## Introduction

Nitrogen (N) is the key nutrient for plant growth and development (Xu *et al.*, 2012). Nitrate transporters are responsible for nitrate uptake from the environment and for internal transport (Y.-Y. Wang *et al.*, 2018). The four gene families involved in nitrate transport comprise NPF (nitrate transporter 1/peptide family), NRT2 (nitrate transporter 2), CLC (chloride channel). and SLAC/SLAH (slow anion channel-associated homologues), and have been reported in Arabidopsis and rice, and were reviewed recently (O’Brien *et al.*, 2016; Fan *et al.*, 2017; H. Li *et al.*, 2017a; Undurraga *et al.*, 2017; Xuan *et al.*, 2017; Tegeder and Masclaux-Daubresse, 2018; Y-Y. Wang *et al.*, 2018). The NPF family includes low-affinity nitrate and peptide transporters sharing high sequence homology and a conserved structural arrangement (Tsay *et al.*, 2007; Léran *et al.*, 2014). The composition of the *NPF* gene family is complex, with between 51 (in *Capsella rubella*) and 139 (in *Malus domestica*) unique members of the *NPF* gene family having been identified in 33 fully sequenced plant genomes, subdivided into eight subfamilies numbered *NPF1*–*NPF8* (Léran *et al.*, 2014).

*NPF* genes have diverse and important functions for nitrogen utilization described in the model plants Arabidopsis and rice (Y.-Y. Wang *et al.*, 2018). *AtNPF6.3* (*CHL1*/*AtNRT1.1*) encodes a dual-affinity (low and high substrate affinity controlled by protein phosphorylation) nitrate transporter, expressed predominantly in roots, and is regulated by N status (Liu *et al.*, 1999; Liu and Tsay, 2003). In addition to roles in nitrate uptake and nitrate translocation from roots to shoots (Tsay *et al.*, 1993; Léran *et al.*, 2013), AtNPF6.3 acts as a nitrate sensor involved in early nitrate signalling of the primary nitrate response (Ho *et al.*, 2009). Subsequently, many other *NPF* genes have been characterized in Arabidopsis and rice, and shown to be involved in different nitrate transport steps in the plant during development (Y.-Y. Wang *et al.*, 2018). In non-plant species, *NPF*-encoded proteins transport di- and tripeptides (Tsay *et al.*, 2007). In Arabidopsis and rice, members of *NPF* subfamilies 7 and 8 have been identified as plasma membrane-located dipeptide transporters involved in dipeptide uptake in roots or pollen tissues, the control of flowering/seed development, and regulating total N content and plant growth (Chiang *et al.*, 2004; Dietrich *et al.*, 2004; Komarova *et al.*, 2008; Fang *et al.*, 2017). Some NPF homologues show chloride or potassium transport activity. AtNPF2.4 loads chloride into the xylem to enable root to shoot chloride transport, and AtNPF2.5 seems to be involved in cortical chloride efflux in the root (B. Li et al., 2016, 2017). AtNPF7.3 may be responsible for proton-coupled potassium loading into the xylem (H. Li *et al.*, 2017b). Although many studies have been conducted on physiological functions of *NPF* genes, little systematic analysis of the *NPF* gene family has been reported, especially in hexaploid wheat (*Triticum aestivum* L.). Previously, wheat *NPF* genes have been identified and partly described, including detailed gene expression analysis (Buchner and Hawkesford, 2014; Bajgain *et al.*, 2018). With the aid of the recently released wheat reference genome (International Wheat Genome Sequencing Consortium, 2018), we carried out a detailed analysis of the complete *NPF* gene family in wheat. This systematic analysis included gene composition, chromosomal locations, and phylogenetic relationships with other plant species including Arabidopsis, *Brachypodium*, and rice. A nomenclature for wheat *NPF* genes is proposed. Further detailed analysis of expression profiles of wheat *NPF* genes was performed using RNA sequencing (RNA-seq) data, as well as using quantitative real-time PCR to investigate responses to nitrogen supply and/or development in different tissues.

## Materials and methods

### Database mining and identification of *NPF* genes in wheat

Protein sequences of four Arabidopsis nitrate transporter gene families, NPF (53 members), NRT2 (7 members), CLC (7 members), and SLAC/SLAH (5 members), were queried based on a blast analysis in InterPro (http://www.ebi.ac.uk/interpro/) for protein domain analysis. A local wheat protein database was established based on the wheat genome (IWGSCv1.1) (https://wheat-urgi.versailles.inra.fr; International Wheat Genome Sequencing Consortium, 2018). Wheat *NPF* gene homologues containing the NPF-specific protein domain of ‘Proton-dependent oligopeptide transporter family’ (IPR000109) HMM profiles (http://www.ebi.ac.uk/interpro/) were identified using HMMER v3.0, with the default parameters and an E-value cut-off of 1e^−5^. A partial domain of IPR000109 and potential false positives were eliminated manually. Candidate *NPF* genes were subjected to analysis for integrities of ORF, protein length, reliability of gene prediction, and sequence redundancy. Homoeologous groups (HGs) were defined by both transcript and protein sequences with >90% (Wan *et al.*, 2017) sequence identities originating from homoeologous chromosomes.

### Phylogenetic analysis

Protein sequence alignments were carried out using MUSCLE (Edgar, 2004) within Geneious® 10.2.3, with default parameters. A phylogenetic tree based on 113 single wheat NPF homoeologues together with 53 Arabidopsis, 75 *Brachypodium*, and 81 rice NPF protein sequences was constructed as described in Wan *et al.* (2017) using PHYML (Guindon *et al.*, 2010) and 100 bootstraps.

### Chromosomal localization of the *NPF* gene family

Physical positions of 331 wheat *NPF* genes were downloaded from URGI (https://wheat-urgi.versailles.inra.fr), and the gene distribution on chromosomes was drawn with MapChart software (Voorrips, 2002) and modified with annotation. Forward and reverse locations of *NPF* genes are indicated by ‘+’ and ‘–’, respectively. Duplicated *NPF* genes were marked with Roman numerals (I, II, III, IV, and V); *TaNPF7.6(1B)* and *TaNPF8.27(7A)* each contains two linked partial genes, annotated with an asterisk.

### Expression analysis of the *NPF* gene family from RNA-seq data

RNA-seq data based on developmental time-course analysis of Chinese Spring (Choulet *et al.*, 2014) and different abiotic and biotic stress experiments were downloaded from the Wheat Expression Browser (www.wheat-expression.com;Ramírez-González *et al.*, 2018). The first study represented five organs (roots, leaves, stem, spikes, and grains) at three developmental stages (two biological replicates). The study on stresses included drought, heat, and drought+heat stress (Liu *et al.*, 2015); cold stress (Li *et al.*, 2015); polyethylene glycol (PEG) stress (N/A); spike drought stress (Ramírez-González *et al.*, 2018); phosphate (Pi) starvation-stressed roots/shoots (Oono *et al.*, 2013); spikelets with *Fusarium*/abscisic acid (ABA)/gibberellin (GA) stress (Buhrow *et al.*, 2016); leaf powdery mildew/stripe rust stress (Zhang *et al.*, 2014); and leaf fungal *Magnaporthe oryzae* stress (Islam *et al.*, 2016). Expression values of *NPF* genes as transcripts per million (tpm) were extracted and summed across homoeologues (see Supplementary Table S6A and B at *JXB* online). The heatmaps (Fig. 3; Supplementary Fig. S3) were constructed by pheatmaps (v1.0.8, Kolde, 2019) and R (v3.5.2) on log_2_-transformed data tpm+1 for 113 *NPF* HGs deduced from 331 *NPF* genes (Supplementary Table S6A, B). Variations of *NPF* gene expressions under abiotic and biotic stresses were listed for analysis by using a threshold of 3-fold changes (Supplementary Table S7).

### Plant material, nitrogen analysis, and RNA extraction

As described previously (Wan *et al.*, 2017), the wheat variety Hereward was grown in field trials in 2015, with 200 kg ha^–1^ (high) or no (low) N application. Roots at Zadoks 23 (Z23; 2–3 tiller stage) and Z45 (booting stage) were excavated from the soil with a garden fork and washed several times using deionized water. Excess water was removed using a soft tissue, and the roots were immediately immersed in liquid nitrogen. Leaves at Z23, Z45, as well as at 5, 14, and 21 dpa (days post-anthesis), stems at Z45, and at 5, 14, 21, and 28 dpa, flag leaf nodes at 5 and 14 dpa, and whole caryopses at 5, 10, 14, 21, and 28 dpa were harvested, freezer milled (Freezer Mill 6870, Spex SamplePrep, Stanmore, UK), and stored at –80 °C for RNA extraction.

Total RNA was isolated by a modified method (Verwoerd *et al.*, 1989) including additional phenol–chloroform–isoamylalcohol extractions. The N concentration of oven-dried subsamples was measured by the Dumas method using a LECO CN628 Combustion Analyser (LECO Corporation, St Joseph, MI, USA) and is expressed in percentage dry matter.

### Reverse transcription–quantitative real-time PCR analysis (RT–qPCR)

First-strand cDNA synthesis was performed using 2 µg of total RNA based on the Invitrogen Superscript III standard protocol. Real-time PCR was performed on an ABI7500 (Applied Biosystems) thermocycler using SYBR^®^ Green JumpStart™ *Taq* ReadyMix™ (Sigma-Aldrich). The 20 μl reactions contained 1 µl of cDNA and 250 nM of each primer. Primer efficiency was analysed and only primer combinations were used with primer efficiencies between 90% and 110%. Primers of *TaNPF2.11(7D)*, *TaNPF5.8(5A)*, *TaNPF5.16(3B)*, *TaNPF7.4(2D)*, and *TaNPF7.7(1B)* were designed according to only one single homoeologue (indicated in parentheses) as no proper common primers for all homoeologues were obtained. Primers of *TaNPF5.9(1)* were designed based on the common sequences of three (*TraesCS5A02G485200*, *TraesCS5B02G498500*, and *TraesCS5D02G498700*) out of six homoeologues. For the remaining *NPF* genes, partly degenerated primers were designed to cover the expression from all the gene homoeologues. *TraesCS4A02G035500/TraesCS4B02G268200/TraesCS4D02G267600* (cell division control protein) was used as the internal control gene for validation in root, leaf, stem, node, and spike (Paolacci *et al.*, 2009), and *TraesCS3A02G186600/TraesCS3B02G216100/TraesCS3D02G190500* (proteasome subunit) was used as the internal control gene for validation in grain. All primer sequences are listed in Supplementary Table S10.

For each primer pair, PCR efficiency was calculated in each run from a pool of all available cDNAs by using LinRegPCR software (Ramakers *et al.*, 2003). All time points had three biological replicates. The normalized relative quantity (NRQ) of expression was calculated in relation to the Ct values and the primer efficiency (E) of both the target gene (X) and reference genes (N) as normalized relative expression (NRE) based on Rieu and Powers (2009): NRE=(E_X_)^–Ct, X^/(E_N_)^–Ct, N^ (all NRE results are in Supplementary Table S11).

Heatmap presentations of RT–qPCR results were constructed for each tissue. All the gene expression data were normalized to the mean expression value of the first detected stage (root/leaf, Z23; stem, Z45; node, 5 dpa; spike, Z45; and grain, 5 dpa) including both low and high N treatments. The heatmaps (Figs 4–8; Supplementary Figs S4–S6) were created by pheatmaps (v1.0.8, Kolde, 2019) and R (v3.5.2) on log_2_-transformed data of normalized data+1.

### Statistics

The statistical validation of the effects of development (D), nitrogen (N), development and nitrogen (D&N) as well as the interaction of development and nitrogen (D–N) on *NPF* gene expression was evaluated on log_2_-transformed NRE and ANOVA using Genstat 18th Edition (VSN International Ltd, UK). Comparisons between relevant means of *n*=3 replicates were made using the error of the difference (SED) on the residual degrees of freedom from the ANOVA, thus invoking the least significant difference (LSD) at the 5% level of significance (pairs of means different by more than the LSD are statistically significantly different, *P*<0.05).

## Results

### Identification of *NPF* genes in wheat

Protein sequence analysis based on the proton-dependent oligopeptide transporter family (IPR000109) protein domain specific for the NPF family (Supplementary Table S1) and the wheat genome database (IWGSCv1.1) initially identified a total of 365 genes including 331 high confidence (HC) and 34 low confidence (LC) genes (Supplementary Table S2). Among these candidates, 15 LC genes lacked start and/or stop codons and 14 genes (4 HC genes and 10 LC genes) encoded very short proteins (< 200 amino acids) which did not meet the multitransmembrane structure of NPF proteins. Three gene sequences (1 unknown chromosomal location HC gene and 2 LC genes) were partial and redundant with another two HC genes, three LC genes had very low gene prediction scores (<30), and one LC gene had an extra stop codon in the coding sequence (Supplementary Table S3). These 34 genes were removed from the candidate *NPF* gene cluster, and 331 *NPF* members including 326 HC and 5 LC genes were classified as wheat *NPF* genes (Supplementary Table S4).

Homoeologous genes were further defined as those with both transcript and protein sequence similarities >90% to corresponding chromosomes in the different subgenomes (Wan *et al.*, 2017), and the majority of homoeologous genes showed >95% similarity. The 331 *NPF* genes were subdivided into 113 *NPF* HGs which contained 73 groups with three, 19 groups with two, seven groups with 4, three groups with 6, one group with 7, and one group with 12 homoeologues, respectively. In addition, there were nine *NPF* genes without any homoeologues in the wheat genome (Supplementary Table S4).

### Phylogenetic analysis of wheat *NPF* genes

**Fig. 1.**
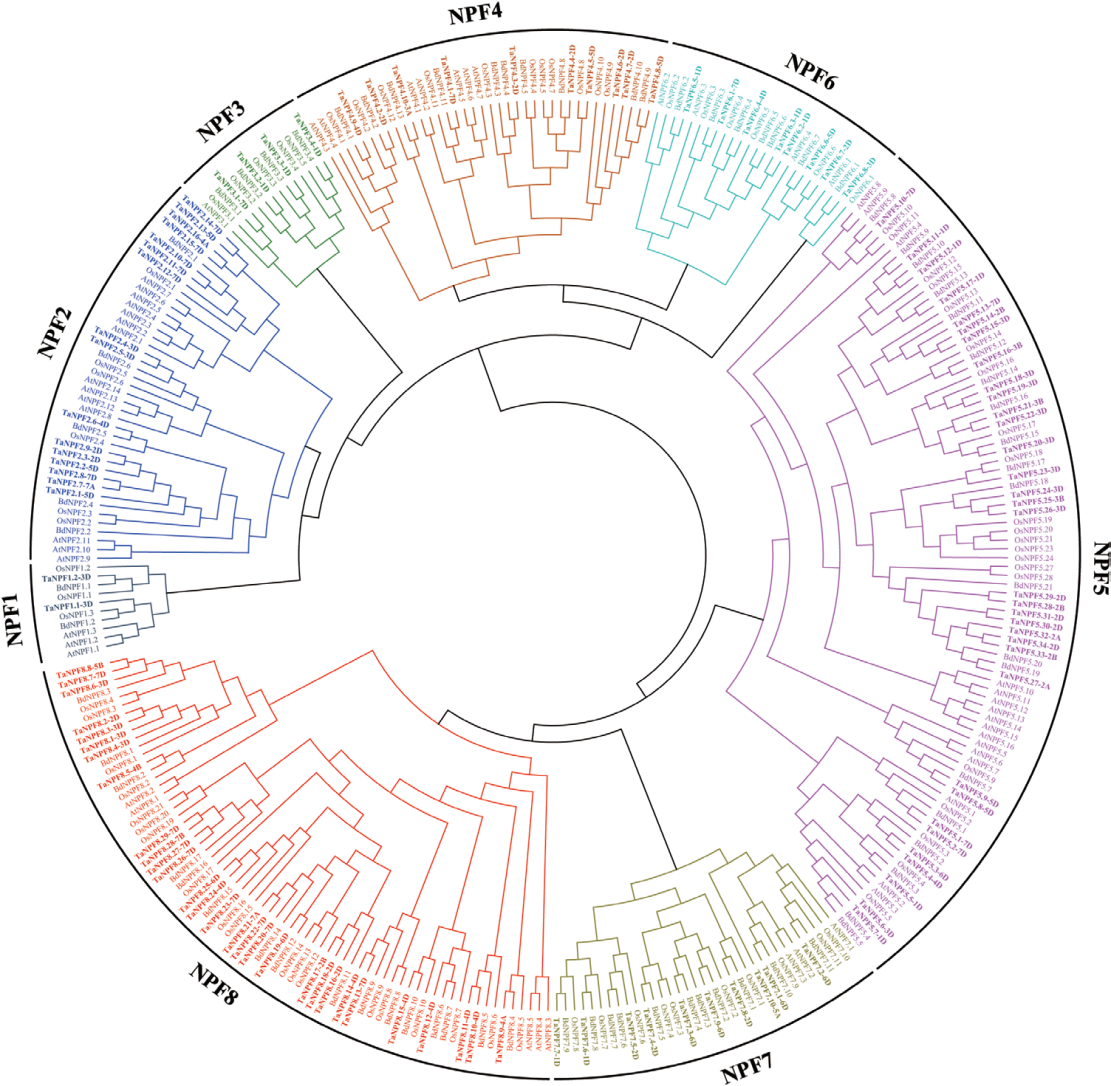
Phylogenetic analysis of the *NPF* gene family of wheat. A total of 113 single wheat *NPF* genes (one from each homeologous group) were aligned with 53 Arabidopsis (*A. thaliana*), 75 *Brachypodium* (*B. distachyon*), and 81 rice (*O. sativa*) *NPF* genes using protein sequences. The tree was constructed using CLUSTALW and PHYML programs in Geneious using the Neighbor–Joining method with 100 bootstrap replicates.

*NPF* genes have been identified in many plants and are classified into eight subfamilies (Léran *et al.*, 2014). For phylogenetic analysis, classification, and systematic nomenclature of wheat *NPF* genes, full-length protein sequences of the 113 single homoeologues from each HG were aligned with the orthologues of Arabidopsis, *Brachypodium*, and rice, and a phylogenetic tree was constructed (Fig. 1). NPF orthologues from Arabidopsis, *Brachypodium*, and rice located together with individual wheat NPF members in the same clades representing the eight subfamilies (NPF1–NPF8). Among the wheat *NPF* gene family, the *NPF5* subfamily contains the most (34 members) *NPF* genes, followed by subfamilies *NPF8* (29 members), *NPF2* (16 members), *NPF4* and *NPF7* (10 members for each subfamily), *NPF6* (8 members), *NPF3* (4 members), and *NPF1* (2 members) with the least *NPF* genes. As 16 wheat *NPF* genes have been previously isolated, numbered, and named (Buchner and Hawkesford, 2014), the other wheat *NPF* genes were further assigned based on their locations and relationships with orthologues of Arabidopsis, *Brachypodium*, and rice with the proposed nomenclature (Léran *et al.*, 2014) (Fig. 1; Supplementary Table S4).

### Chromosomal locations of wheat *NPF* genes

**Fig. 2.**
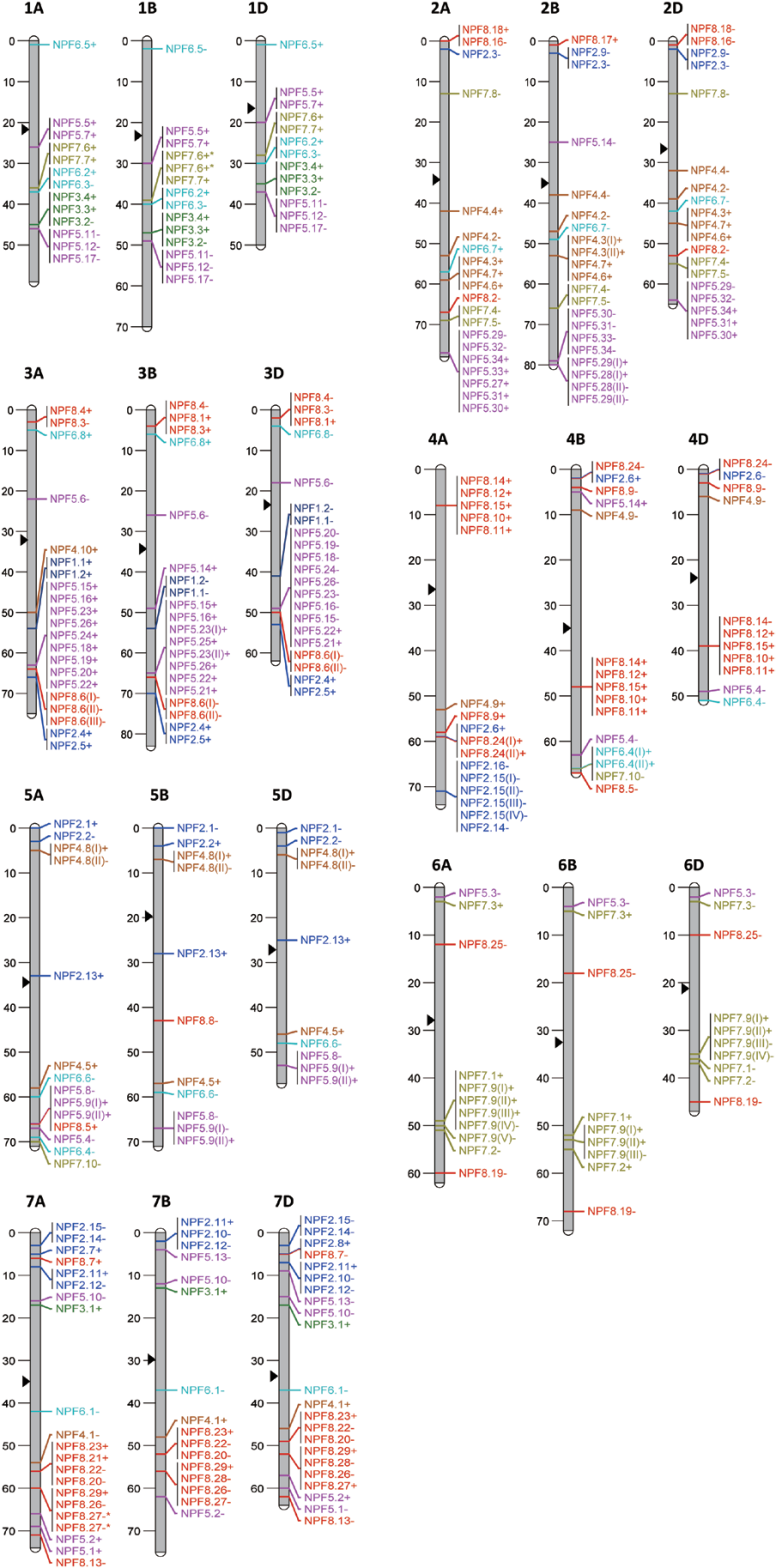
Distribution of *NPF* genes on wheat chromosomes. The centromeres are indicated by arrowheads.

To determine the chromosomal distributions of *NPF* genes, 328 out of 331 genes were mapped on the wheat chromosomes using physical positions (Fig. 2; Supplementary Table S5). Chromosomes 3, 2, and 7 have the most abundant *NPF* genes (62, 60, and 59 members, respectively), followed by chromosomes 4, 1, and 5 (42, 40, and 35 members, respectively), while chromosome 6 has the least *NPF* genes (30 members; Fig. 2). Three *NPF* genes, *TaNPF7.10* (*TraesCSU01G130200*), *TaNPF8.5* (*TraesCSU01G115500*), and *TaNPF8.18* (*TraesCSU01G307300LC*), are probably located on Chr4D, Chr4D, and Chr2B, respectively, according to the locations of the respective homoeologues (Supplementary Table S4). The majority of *NPF* genes are located distant from the middle of the chromosomes, and only a few genes are present near to the centromere (Fig. 2). On each chromosome, more *NPF* genes are located on the long arm compared with the short arm of the chromosome (Fig. 2).

### Expression patterns and validation of wheat *NPF* genes

**Fig. 3.**
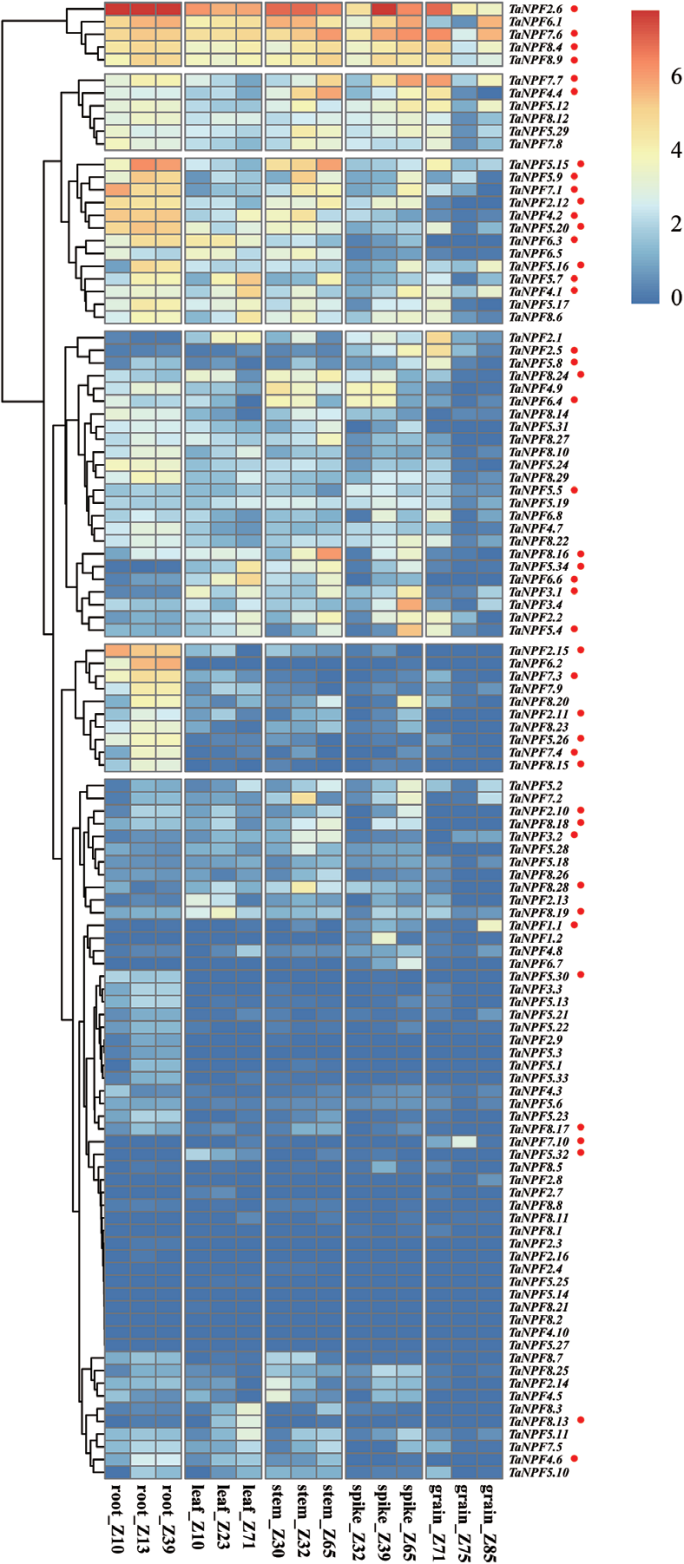
Heatmap of spatio-temporal expression profiles of wheat *NPF* genes based on RNA-seq data (Choulet *et al.*, 2014). Zadoks (Z) developmental stages are shown. Z10, seedling stage; Z13, three leaf stage; Z23, tillering stage; Z30, 1 cm spike; Z32, two nodes detectable; Z39, flag leaf stage; Z65, anthesis; Z71, 2 d post-anthesis; Z75, 14 d post-anthesis; Z85, 30 d post-anthesis. The different colours represent the abundances of the transcripts based on log_2_-transformed tpm (transcript per million)+1. Red dots marked *TaNPF* genes selected for RT–qPCR gene expression analysis.

To gain insight into the spatial and temporal expression patterns of *NPF* genes in wheat, the RNA-seq data sets derived from root, leaf, stem, spike, and grain of the wheat cultivar Chinese Spring (Choulet *et al.*, 2014) were explored. The possible interactions of *NPF* gene expression in relation to biotic and abiotic stresses were verified by analysis of RNA-seq data sets derived from different stress experiments (Ramírez-González *et al.*, 2018). The spatio-temporal expression profiles of *NPF* genes were clustered into six groups (Fig. 3) with no specific relationship to the wheat *NPF* phylogenetic subfamily structure. *NPF* genes in group I were highly expressed in almost all tissues, with *TaNPF2.6* the most abundantly expressed gene. In groups II and III, the majority of the *NPF* genes were expressed at medium to high levels. In general, the expression of group II *NPF* genes in stems, spikes, and grains depended on the growth stage, whereas expression in roots was relatively constant. The majority of group III *NPF* genes had relatively high expression levels in roots, and lower expression in stems and leaves. Most *NPF* genes in group IV were expressed at intermediate levels in all tissues. Interestingly, all *NPF* genes in group V were expressed mainly in the roots, indicating root-specific transport functions. Group VI included most of the *NPF* genes with a less complex tissue specificity pattern and typically low or extremely low expression levels (Fig. 3).

Selected spatio-temporal RNA-seq data were verified by RT–qPCR for eight NPF genes from the six expression groups in roots, leaves, stems, and grain for the wheat cultivar Hereward. The results of the real-time expression analysis are in agreement with the RNA-seq data, except for *TaNPF1.1*, which had relatively high expression levels in roots in addition to in the grain (Supplementary Figs S1, S2).

The stress-related RNA-seq expression analysis was clustered in relation to the phylogenetic *NPF* subfamily structure (Supplementary Fig. S3). The majority of the *NPF* HGs reacted specifically to different stresses. When using a minimum of a 3-fold change as a threshold, 21 out of the 113 HGs did not show any changes of gene expression (Supplementary Table S7). There was no obvious correlation of specific stress-related changes of *NPF* HG expression in relation to the phylogenetic *NPF* subfamily structure (Supplementary Fig. S3; Supplementary Table S7). Stress-related up- or down-regulation of members of all *NPF* subfamilies were seen, apart from leaf heat stress (*NPF1*), leaf PEG stress (*NPF1* and *NPF3*), spike drought stress (*NPF3* and *NPF6*), phosphate starvation (*NPF1*, *NPF2*, *NPF4*, and *NPF8*), leaf stripe rust pathogen inoculation (*NPF1*, *NPF3*, and *NPF6*), and leaf *M. oryzae* inoculation (*NPF1*, *NPF3*, *NPF4*, and *NPF6*). Gene expression of 38 HGs representing all *NPF* subfamilies was changed specifically by abiotic stresses, and only eight *NPF* HGs from *NPF2*, *NPF5*, *NPF7*, and *NPF8* subfamilies were changed solely by biotic stress. Leaf heat and drought stress resulted in the strongest response of the whole *NPF* gene family, where the majority of *NPF* HGs were down-regulated (heat, 44 members; drought, 25 members; heat+drought, 55 members). The spike response to drought comprised 8 up- and 13 down-regulated *NPF* HGs, with lower intensity compared with leaves. A larger number of *NPF* HGs were up-regulated rather than down-regulated in cold-treated shoots and PEG-treated leaves. Biotic-related stresses such as *Fusarium*/ABA/GA treatments, leaf powdery mildew, and leaf strip rust resulted in an equal number of *NPF* HGs up- or down-regulated, although for fungal stresses more *NPF* HGs were down-regulated. Only two members of *NPF* subfamily 1 were mostly up-regulated under different stresses: *TaNPF1.1* responded to cold, *Fusarium*/ABA, and powdery mildew stress, and *TaNPF1.2* in an opposite way for drought in leaves and spikes. The majority of *NPF* genes reacted to both abiotic and biotic stresses and some were regulated in an opposite way under different stresses. Some *NPF* genes which were down-regulated by drought/heat stress were up-regulated by cold stress. Some members of *NPF* subfamily 7 reacted with a strong up-regulation under most biotic stresses in contrast to their reaction to abiotic stresses. The same was seen for some members of *NPF* subfamilies 5 and 8. A very small number of *NPF* HGs responded to phosphate starvation in roots and shoot: two *NPF* subfamily 5 HGs were down-regulated in roots, whereas in shoots up- and down-regulation were seen for four *NPF* subfamily HGs (Supplementary Table S7).

### *NPF* gene expression profiles under nitrogen treatments

Most *NPF* genes are linked to N acquisition and utilization, as indicated by their respective substrates. To determine *NPF* gene expression responses to N supply during development, 44 *NPF* genes were selected according to their expression patterns from the RNA-seq data (Fig. 3), with the feature of being highly expressed in tissues, or preferred expression in a specific tissue at different expression levels (for details of the selection, see Supplementary Protocol S1). These selected *NPF* genes represented the eight phylogenetic subfamilies and the six *NPF* family gene expression groups (Figs 1, 3). After further subselection based on the tissue distribution of the six expression groups (Supplementary Protocol S1), their gene expression patterns were analysed in roots, leaves, stems, nodes, spikes, and developing grains at different growth stages cultivated under high and low N fertilizer treatments in field experiments. Zero N fertilization had a strong influence on plant performance in comparison with 200 kg N ha^–1^. Grain and straw yields were reduced by 58% and 70%, respectively, with grain and straw N contents reduced by 30% and 76%, respectively. N deficiency was indicated by a drastically reduced grain and straw N accumulation (70% and 78%), with a harvest index slightly higher under the zero N treatment (Table 1). The effects of development, N, and interaction of development and N on gene expression were analysed.

**Table 1.** Field performance of wheat cv. Hereward in relation to reduced nitrogen fertilization

N rate (kg ha^–1^)	Grain yield (t ha^–1^, 85% DM)	Straw yield (t ha^–1^, 85% DM)	Grain % N	Straw % N	Grain N accumulation (kg ha^–1^)	Straw N accumulation (kg ha^–1^)	Harvest index
0	4.84±0.2	2.89±0.5	1.33±0.008	0.256±0.006	54.91±2.9	6.21±0.9	63.56±3.7
200	11.24±0.12	9.42±1.0	1.84±0.01	0.354±0.01	176.13 ±3.3	28.54±3.9	54.82±2.6

### Root *NPF* gene expression

**Fig. 4.**
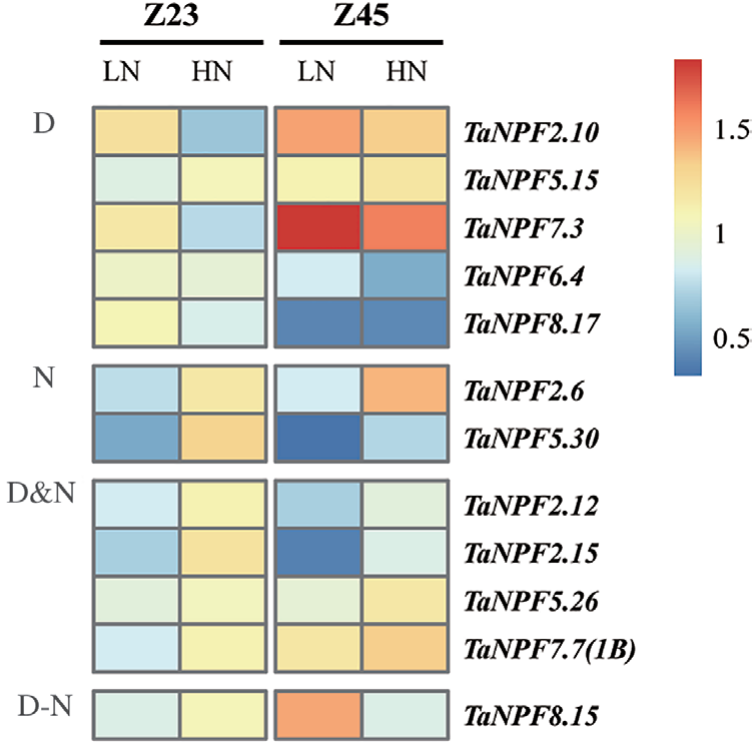
Heatmap of *NPF* expression profiles in root in relation to N fertilization and development by RT–qPCR. Gene expression data were normalized for each gene and are shown as log_2_-transformed data of normalized data+1. Z23, tillering stage; Z45, booting stage; LN, low nitrogen (0 kg ha^–1^) application; HN, high nitrogen (200 kg ha^–1^) application. Groups D, N, D&N, and D–N indicate development effect (D), nitrogen effect (N), development and nitrogen effects (D&N), and interacting effects of development and nitrogen (D–N) on *NPF* gene expression (*n*=3 replicates; *P*<0.05), respectively.

The root plays roles in initial N acquisition. Previously, no *NPF* root expression of field-grown root samples has been analysed. As root N uptake depends on plant development, root gene expression was analysed at two growth stages, Z23 (2–3 tiller stage) and Z5 (booting stage) (Fig. 4; Supplementary Fig. S4). Expression of the majority of the 28 subselected *NPF* genes were not affected by development or N supply (Supplementary Fig. S4). However, the expression of seven *NPF* genes was significantly affected by N supply (Fig. 4). For six genes, transcript levels were decreased by low N (Fig. 4), and four of these *NPF* genes were also developmentally up- or down-regulated at the two growth stages (Fig. 4). The expression of *TaNPF8.15* showed an interaction between N treatment and development, with a significant increase of transcript abundance at the booting stage at low N supply (Fig.4). Five *NPF* genes were developmentally regulated, with three and two *NPF* genes significantly up-regulated or down-regulated, respectively, from Z23 to Z45 (Fig. 4).

### Leaf *NPF* gene expression

**Fig. 5.**
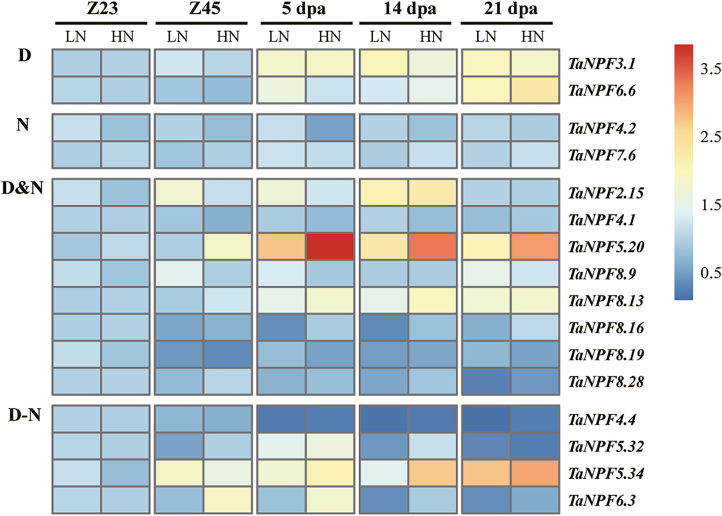
Heatmap of *NPF* expression profiles in the flag leaf in relation to development and N fertilization by RT–qPCR. Gene expression data were normalized for each gene and are shown as log_2_-transformed data of normalized data+1. Z23, tillering stage; Z45, booting stage; dpa, days post-anthesis; LN, low (0 kg ha^–1^) nitrogen application; HN, high (200 kg ha^–1^) nitrogen application. Groups D, N, D&N, and D–N indicate development effect (D), nitrogen effect (N), development and nitrogen effects (D&N), and interacting effects of development and nitrogen (D–N) on *NPF* gene expression (*n*=3 replicates; *P*<0.05), respectively.

Depending on the plant growth stage, leaves may be N sinks or N sources, both of which involve N allocation and redistribution. The gene expression patterns of 16 selected *NPF* genes (Supplementary Protocol S1) were analysed in flag leaves at different growth stages (Z23 and Z45; and 5, 14, and 21 dpa) (Fig. 5). The transcript abundances of 14 of these *NPF* genes were influenced by the N fertilization and resulted in increased or reduced gene expression with additional or interactive developmental effects in both up and down directions (Fig. 5). For just two *NPF* genes (Fig. 5), N fertilization had no effect on the expression pattern, but both genes were up-regulated post-anthesis.

### Stem *NPF* gene expression

**Fig. 6.**
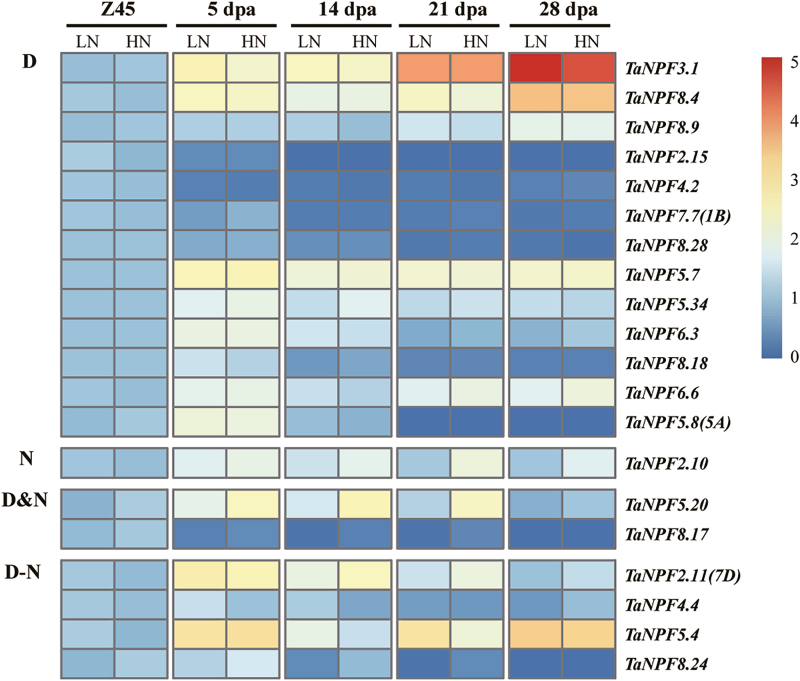
Heatmap of *NPF* expression profiles in the stem in relation to development and N fertilization by RT–qPCR. Gene expression data were normalized for each gene and are shown as log_2_-transformed data of normalized data+1. Z45, booting stage; dpa, days post-anthesis; LN, low nitrogen (0 kg ha^–1^) application; HN, high nitrogen (200 kg ha^–1^) application. Groups D, N, D&N, and D–N indicate development effect (D), nitrogen effect (N), development and nitrogen effects (D&N), and interacting effects of development and nitrogen (D–N) on *NPF* gene expression (*n*=3 replicates; *P*<0.05), respectively.

The stem is the conduit for N compound translocation. Gene expression responses to N supply from Z45 to 28 dpa were analysed for 20 subselected *NPF* genes (Fig. 6). Seven *NPF* genes were found to be responsive to N supply, mainly by down-regulation under low N (Fig. 6). For *TaNPF4.4*, low N initiated a post-anthesis increase of transcript which diminished until ripening, whereas *TaNPF5.4* was more highly expressed in the post-anthesis period under low N compared with sufficient N. For most of the *NPF* genes, gene expression in the stem was influenced by development with three typical patterns (Fig. 6): a steady post-anthesis increase or decrease of transcript, or a short increase of expression between Z45 and 5 dpa, followed by steady state or declining levels.

### Node *NPF* gene expression

**Fig. 7.**
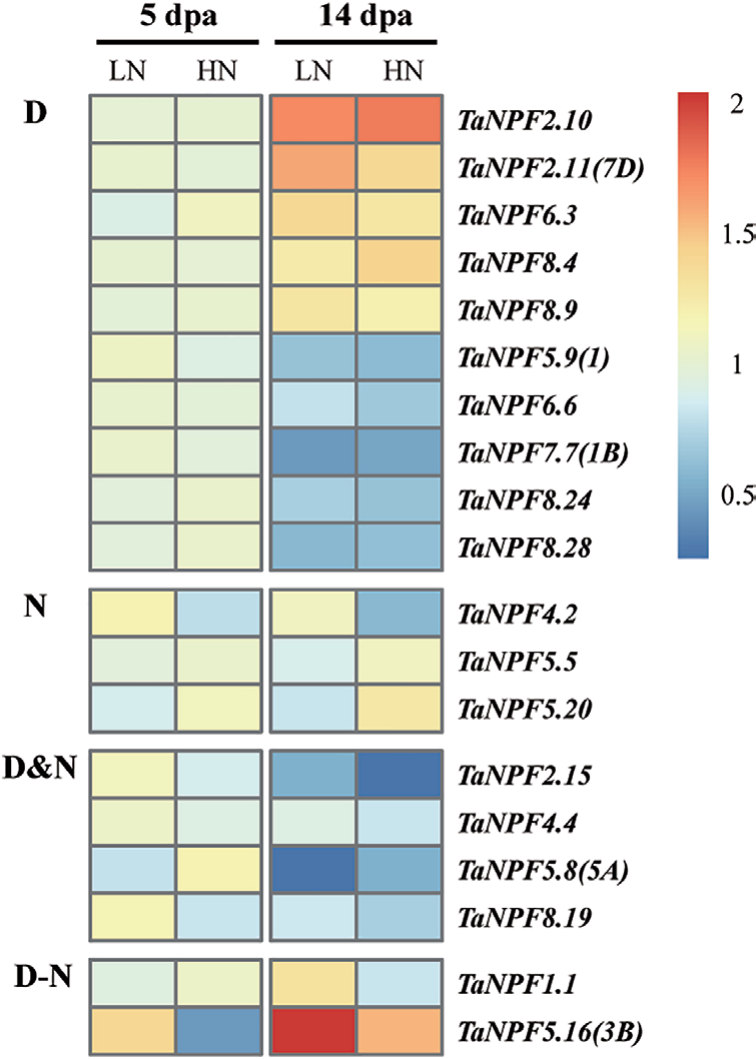
Heatmap of *NPF* expression profiles in the node in relation to development and N fertilization by RT–qPCR. Gene expression data were normalized for each gene and are shown as log_2_-transformed data of normalized data+1. dpa, days post-anthesis, LN, low nitrogen (0 kg ha^–1^) application; HN, high nitrogen (200 kg ha^–1^) application. Groups D, N, D&N, and D–N indicate development effect (D), nitrogen effect (N), development and nitrogen effects (D&N), and interacting effects of development and nitrogen (D–N) on *NPF* gene expression (*n*=3 replicates; *P*<0.05), respectively.

The node is the junction of stem, leaf, and axillary bud/tiller, and possesses well-developed vascular bundles in Poaceae such as rice, barley, and wheat (Yamaji and Ma, 2017). Twenty-seven *NPF* genes were selected (Supplementary Protocol S1) for analysis of gene expression in the flag leaf node at 5 and 14 dpa (Fig. 7; Supplementary Fig. S5). Expression levels of three of the nine N-responsive *NPF* genes were not developmentally influenced (Fig. 7). The other *NPF* genes were additionally developmentally down-regulated between both time points (Fig. 7). Some significant N–development interactions were seen: *TaNPF5.16* was up-regulated under both N conditions, whereas for *TaNPF1.1* only low N resulted in a significant increase of transcript abundance at 14 dpa (Fig. 7). Ten *NPF* genes were responsive to node development only, with increased or reduced transcript abundance between 5 and 14 dpa (Fig. 7). Eight *NPF* genes were not significantly responsive to either N supply or development (Supplementary Fig. S5).

### Spike *NPF* gene expression

Expression levels of 20 selected *NPF* genes (Supplementary Protocol S1) were analysed in spikes at Z45 stage. As shown in Supplementary Fig. S6, none of the genes showed significant effects of N treatments, indicating consistent expression of *NPF* genes in the young reproductive organs.

### Grain *NPF* gene expression

**Fig. 8.**
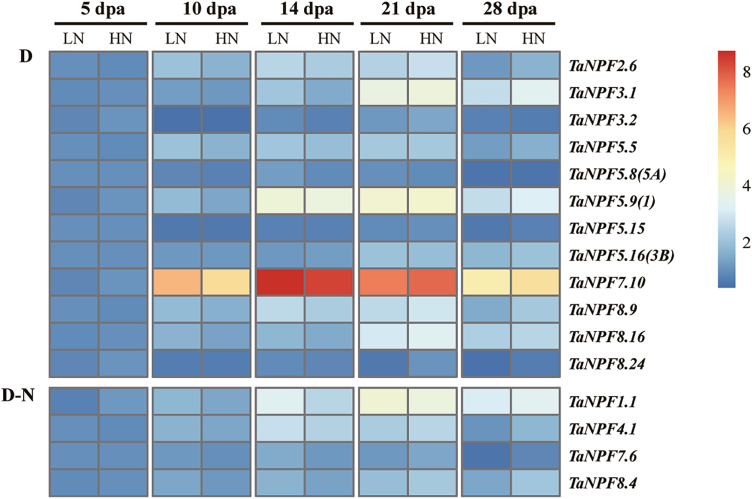
Heatmap of *NPF* expression profiles in the grain by RT–qPCR. Gene expression data were normalized for each gene and are shown as log_2_-transformed data of normalized data+1. dpa, days post-anthesis, LN, low nitrogen (0 kg ha^–1^) application; HN, high nitrogen (200 kg ha^–1^) application. Groups D, N, D&N, and D–N indicate development effect (D), nitrogen effect (N), development and nitrogen effects (D&N), and interacting effects of development and nitrogen (D–N) on *NPF* gene expression (*n*=3 replicates; *P*<0.05), respectively.

During post-anthesis development, grains are the main sinks for N, originating particularly from redistribution from vegetative organs (leaf, stem, etc.). The expression of 16 *NPF* genes was analysed in developing grains between 5 and 28 dpa (Fig. 8). Gene expression in the grain of four *NPF* genes responded to N fertilization with a specific developmental pattern (Fig. 8). During early to mid-grain filling, between 5 and 14/21 dpa, there was an increase of transcript levels of the four *NPF* genes, with higher expression levels at low N. With further grain development, gene expression of *NPF* genes decreased rapidly under low N, with no or reduced change under sufficient N supply. This overall post-anthesis developmental pattern was very similar for nearly all 16 *NPF* genes for both N treatments (Fig. 8).

## Discussion

### Composition of the *NPF* gene family in wheat

To date, the composition of *NPF* gene families has been identified for >30 plants (Léran *et al.*, 2014). In wheat, *NPF* genes were previously described (Buchner and Hawkesford, 2014; Bajgain *et al.*, 2018). The completeness of the newly released wheat genome data (International Wheat Genome Sequencing Consortium, 2018) allowed the identification of 331 non-redundant *NPF* genes (Supplementary Table S4). Among the wheat *NPF* gene family, five low confidence (LC) genes were included due to genome sequence gene structure re-validation and confirmed expression signals in RNA-seq data. Recently, a pseudo reference consisting of 500 potential wheat *NRT* (nitrate transporter) gene sequences was established for wheat seedling RNA-seq expression analysis (Bajgain *et al* 2018). After re-analysis of the 500 gene IDs based on the TGAC (The Genome Analysis Centre) 2015 assembly annotation in comparison with the current wheat genome IWGSCv1.1 database, we identified just 270 individual *NRT* genes. Some of the 500 gene IDs mapped as duplicates and not as individual *NRT* genes. Additionally, some of the provided gene IDs do not represent individual *NRT* genes or are mapped on different chromosomes indicated by the former TGAC assembly. Some of the genes represent HGs. Finally, 234 genes of the study corresponded to unique *NPF* genes (Bajgain *et al.*, 2018) (Supplementary Table S8). The remaining 36 *NRT* genes included 28 clade 5 *NRT2* family and eight clade 1 *NAR2* family genes (Bajgain *et al.*, 2018) (Supplementary Tables S1, S9). The present analysis identified 97 new *NPF* genes to complete the whole *NPF* gene family in wheat, which contains more genes than the other three nitrate transporter gene families, *NRT2*, *CLC*, and *SLAC/SLAH* (Krapp *et al.*, 2014; Fan *et al.*, 2017; H. Li *et al.*, 2017a; Y.-Y. Wang *et al.*, 2018). The chromosomal locations of the wheat *NPF* genes are unevenly distributed, with high gene abundances in chromosomes 2, 3, and 7 (Fig. 2). This is also the case for members of another large N-related gene family in wheat, the amino acid transporter (AAT) family (Wan *et al.*, 2017). Whereas the *AAT* genes are mostly located close to the centromere of each chromosome (Wan *et al.*, 2017), the majority of the *NPF* genes are located distant from the centromere, with a favoured distribution on the long arm of the chromosomes (Fig. 2).

The wheat *NPF* gene family represents one of the largest *NPF* gene families among the currently analysed species (Léran *et al.*, 2014). Theoretically, every wheat gene would have three homoeologous genes as the result of allohexaploidization, but many wheat *NPF* genes have no or only one homoeologous gene, resulting from the side effects of polyploidy, and evolutionary and acclimation processes (Lynch and Force, 2000). The 331 wheat *NPF* genes were subdivided into 113 HGs (Supplementary Table S4). In addition to 73 (64.6% of the total) groups with three HGs, 19 (16.8%) groups contained two HGs and nine (8.0%) groups were singleton genes without any homoeologues. Gene duplication is a common phenomenon contributing to gene family expansion (Zhang, 2003), and they are commonly detected in wheat interspecific whole-genome analysis (International Wheat Genome Sequencing Consortium, 2014). For the wheat *NPF* family, seven (6.2%), three (2.7%), one (0.9%), and one (0.9%) HGs contained four, six, seven, and as many as 12 homoeologues, respectively (Supplementary Table S4). The group with 12 homoeologous genes was composed of five, three, and four neighbour-linked duplicated genes located on Chr6A, Chr6B, and Chr6D, respectively. The results revealed that the composition of the wheat *NPF* gene family is much more complex than that in most other plant species. The complexity of the wheat *NPF* gene family results mainly from integration of normally three homoeologues and additional gene duplication and/or loss of genes during wheat evolution. This complexity does not influence their phylogeny relationships with orthologues in other plants. The 113 wheat *NPF* HGs could be classified into eight clades in the phylogenetic tree, similarly to the *NPF* genes in other plant species (Fig. 1; Supplementary Table S4), providing the basis for a systematic nomenclature of wheat *NPF* genes (*TaNPFX.Y*) as proposed, except for the 16 *NPF* genes already named previously (Buchner and Hawkesford, 2014; Léran *et al.*, 2014). Each clade shows further subtree structure leading to direct and indirect orthologous relationships between the plant species.

### Diverse wheat *NPF* gene expression patterns and responses to abiotic and biotic stresses

In addition to nitrate and peptides, NPF transporters have diverse and mixed substrate specificities including ions (chloride, nitrite), organic compounds (glucosinolates, dimethylarsinate), and hormones (auxin, ABA, jasmonates, and GAs) (Corratgé-Faillie and Lacombe, 2017; Y.-Y. Wang *et al.*, 2018), indicating the broad participation of *NPF* genes in biological processes involved in adaptation to adverse environmental conditions. Apart from N starvation, some studies have shown specific *NPF* gene responses to abiotic and biotic stresses (Pike *et al.*, 2014; Taochy *et al.*, 2015; Zhang *et al.*, 2018). The RNA-seq analysis in relation to different abiotic and biotic stresses in wheat illustrates a complex pattern throughout the whole *NPF* gene family (Supplementary Fig. S3; Supplementary Table S7). These patterns did not show any correlation to the phylogenetic subfamily structure of the wheat *NPF* gene family. A mixed pattern of up- or down-regulation of *NPF* genes indicated multiple functions of the *NPF* family in response to most of the abiotic and biotic stresses. The strongest response was observed for drought/heat stress, for which 61 of the 113 HGs were affected with 55 HGs down-regulated by the stress (Supplementary Table S7). A major consequence of leaf drought stress is an imbalance of carbon and N metabolism due to a decrease in photosynthesis with a strong effect on carbon and N compound translocation (Yang *et al.*, 2019). The wide responses of *NPF* genes to drought suggest a strong down-regulation of N substrate transport in leaves, reflecting the slow down of N metabolism under drought stress. However, the drought response of *NPF* genes was not so drastic in spikes (22 *NPF* genes) with partly opposite up or down gene regulation in response to the stress compared with leaves, indicating a different regulation mechanism in the reproductive organ. Co-application of *Fusarium* stress and ABA to wheat spikelets resulted in ABA-specific up- or down-regulation of different *NPF* genes (Supplementary Table S7). Interestingly, most of the ABA-responsive wheat *NPF* genes were also influenced by heat and/or drought and/or cold stresses. ABA plays an important role in abiotic stress response and tolerance by regulating stomatal response and stress-related gene expression (Chinnusamy *et al.*, 2008). The broad responses of *NPF* genes to ABA and abiotic stresses suggest that these wheat *NPF* genes are possibly involved in ABA transport and/or are hormonally regulated. The response of co-application of *Fusarium* with GA to spikelets induced only three *NPF* genes, *TaNPF3.4*, *TaNPF5.3*, and *TaNPF7.10. TaNPF3.4* may play an important role in abiotic/biotic stress responses, as it is also highly up-regulated by co-application of *Fusarium* with ABA, as well as by heat, drought, and cold stresses (Supplementary Table S7). The only *NPF3* orthologue in Arabidopsis, *AtNPF3.1*, in addition to nitrate/nitrite transport, also transports and is transcriptionally regulated by both GA and ABA (Pike *et al.*, 2014; Tal *et al.*, 2016), suggesting a similar function for the wheat *TaNPF3.4*. In grapevine and Arabidopsis, *NPF3* subfamily genes are induced by leaf powdery mildew infection (Pike *et al.*, 2014), which was not observed for orthologous wheat *NPF3* genes (Supplementary Table S7). However, *NPF* genes of other subfamilies, especially *NPF7*, showed a strong up-regulation by the fungal stress (Supplementary Table S7). In comparison, *NPF5* subfamily genes showed a strong down-regulation by the fungal stress (Supplementary Table S7). In addition, *NPF2*, *NPF5*, and *NPF8* subfamilies showed more reaction to leaf stripe rust and *M. oryzae*, compared with other subfamilies (Supplementary Table S7). These results indicated the specific roles of individuals from these subfamilies in fungal stress responses. In general, the stress analysis of wheat *NPF* family genes revealed a complex regulation with a mix of common or opposite regulation of genes under different abiotic and biotic stresses with additional components of tissue and developmental regulation of the stress response. Together with the diverse and mixed substrate specificities in addition to nitrate and peptides, these results strengthen the hypothesis that *NPF* members are the basis of the integration of environmental and physiological information linked to the relative availability of nutrients (Corratgé-Faillie and Lacombe, 2017).

### Wheat *NPF* genes are regulated by nitrogen fertilization and/or development

The further validation of *NPF* gene expression response to N fertilization by RT–qPCR demonstrated the complex expression pattern of the wheat *NPF* gene family. Expression of a total of 44 pre-selected *NPF* genes based on phylogeny and the RNA-seq analysis was monitored in root, leaf, stem, node, spike, and grain samples (Figs 4–8; Supplementary Figs S4–S6). This analysis indicated that the expression of *NPF* genes in wheat showed dynamic variations throughout development and in response to available N supply. In summary, tissue-, development-, and N supply-related expression patterns identified six general expression groups. In roots, expression of 16 out of 28 analysed *NPF* genes did not change in relation to N fertilization and development (Supplementary Fig. S4), and most had stable expression levels in RNA-seq analysis of root samples (Fig. 3), suggesting a constitutive function in N homeostasis and root growth. The other *NPF* genes were up-regulated under high N fertilizer and/or by developmental stages (Fig. 4). The up-regulation of *NPF* genes by N provision in roots probably facilitates N uptake and/or translocation from root to shoot as seen in Arabidopsis and rice (Supplementary Table S12). The developmental up- or down-regulation of *NPF* genes may be related to the drastically contrasting demands for root N uptake, translocation, and growth between the Z23 stage (2–3 tillers) and Z45 stage (up to 6 tillers). The nodes are the hub for nutrient distribution in graminaceous plants with their complex vascular system (Yamaji and Ma, 2014, 2017). The development- and/or N-regulated expression of as many as 19 out of 27 *NPF* genes found in nodes suggests a strong participation in transport and regulatory processes of N compound delivery to the reproductive tissues/organs (Fig. 7). The stem mediates transport between root, leaves, and reproductive tissue/organs in varying directions depending on the developmental demand. The expression patterns of *NPF* genes in the stem from the pre-anthesis vegetative stage to the post-anthesis reproductive stage were mostly characterized by increasing or decreasing transcript levels throughout the period, or peaking at the beginning of grain development (5 dpa), followed by reduction until complete ripening (Fig. 6). These expression patterns indicate that the demand for individual *NPF* genes in the stem depends on development, and may involve different transport actions/directions which have to ensure N compound delivery for grain development with N accumulation. The expression of *NPF* genes in leaves also followed the vegetative/reproductive transition. Similarly, to the stem, most *NPF* gene expression in leaves showed developmental regulation, with increasing, decreasing, or a mixed expression pattern with development (Fig. 5). The analysis revealed that *NPF* gene expression was more responsive to N supply in flag leaves compared with other organs. With the exception of *TaNPF3.1* and *TaNPF6.6*, all the 14 other *NPF* genes had increased or decreased expression in response to N fertilization, with partial additional or interactive developmental effects in both up and down directions. Generally, leaves are important N sinks during vegetative growth and convert to N sources during reproductive growth (Tegeder and Masclaux-Daubresse, 2018). The present results indicate participation of *NPF* genes in leaf N compound allocation and remobilization, respectively, depending on the plant growth stage. In comparison, the expression of *NPF* genes in reproductive tissues (spike and grain) was not influenced by N supply as much as in vegetative tissues (Fig. 8; Supplementary Fig. S6). With the exception of four *NPF* genes (*TaNPF1.1*, *TaNPF4.1*, *TaNPF7.6*, and *TaNPF8.4*) in grains showing interactive regulation by N and development, none of the other *NPF* genes showed responses to N supply in spikes and grains.

### Orthologues and NPF functions

*NPF* genes play fundamental roles and participate widely in the complex processes of N utilization (Y.-Y. Wang *et al.*, 2018). There are functional data of 38 and 17 characterized *NPF* genes in Arabidopsis and rice, respectively (Supplementary Table S12). Putative functions in N utilization of the individual wheat *NPF* genes may be deduced by linking the wheat analysis with the orthologues reported in Arabidopsis and rice. The phylogenetic analysis of the wheat NPFs revealed multiple orthologous relationships to Arabidopsis and rice, with direct orthologous genes present in the same subtree and indirect orthologous relationships present in the same NPF subfamily (Fig. 1). *AtNPF6.3* (*NRT1.1*) was the first identified nitrate transporter, functioning in nitrate uptake in root, nitrate translocation from root to shoot, and as a nitrate transceptor to govern many molecular, physiological, and morphological responses to nitrate (Tsay *et al.*, 1993; Liu *et al.*, 1999; Ho *et al.*, 2009; Léran *et al.*, 2013; Bouguyon *et al.*, 2015; Supplementary Table S12). Two rice orthologues of *NRT1.1*, *OsNPF6.5* and *OsNPF6.3*, have diverged in subcellular location, and N (nitrate/ammonium) response and utilization, but both showed potential for improving N use efficency (NUE) and yield of rice (Hu *et al.*, 2015; W. Wang *et al.*, 2018; Supplementary Table S12). The two maize orthologues of *NRT1.1*, *ZmNPF6.6* and *ZmNPF6.4*, showed different substrate preferences and different expression responses to N supply, and were used to improve NUE of maize (Allen *et al.*, 2016; Wen *et al.*, 2017). There are four *NRT1.1* orthologues in wheat, which showed varied expression patterns in RNA-seq and/or RT–qPCR analysis (Figs 1, 3; Supplementary Table S4; Buchner *et al.*, 2014). *TaNPF6.1* was one of the most highly expressed genes of group I, with constitutive expression in different tissues (Fig. 3). *TaNPF6.2* was predominantly expressed in roots, while *TaNPF6.3* was mainly expressed in root, leaf, and stem, and was up-regulated by high N supply in leaves but not in stems and nodes (Figs 3, 5–7). *TaNPF6.4* showed preferred expression in spike and node, and a lower expression in root and leaf (Fig. 3). The expansion in gene number and variation in expression patterns suggests the divergence of *NRT1.1* orthologues in wheat and indicates possible important roles in NUE as previously reported.

Rice *OsNPF2.4* mediates not only nitrate acquisition, but also root to shoot nitrate transport and N remobilization from source to sink organs (Xia *et al.*, 2015). *OsNPF2.4* (also *OsNPF6.5*) was discovered by a genome-wide association study (GWAS) on NUE-related agronomic traits (Tang *et al.*, 2019). Wheat *TaNPF2.6* is the closest orthologue of *OsNPF2.4* and shares high sequence identity (87%) with *OsNPF2.4* (Fig. 1; Supplementary Table S12). Interestingly, *TaNPF2.6* is the most highly expressed gene among the 113 *NPF* genes in wheat (Fig. 3 ; Supplementary Fig. S3), and was induced by N supply in roots (Fig. 4). Whether *TaNPF2.6* plays the important roles in wheat N utilization as seen for *OsNPF2.4* in rice needs to be characterized in the future.

Recently, members of *NPF7* subfamily genes, *OsNPF7.1*–*OsNPF7.4* and *OsNPF7.7*, were reported to be involved in N allocation and shown to have specific roles in regulating tiller number and subsequently the grain yield of rice (Hu *et al.*, 2016; Fang *et al.*, 2017; Huang et al., 2018, 2019; J. Wang *et al.*, 2018; Supplementary Table S12). In wheat, there are 10 *NPF7* members (*TaNPF7.1*–*TaNPF7.10*) which showed variable expression patterns (Figs 1, 3; Supplementary Table S4). Further detection by RT–qPCR revealed that the expression patterns of some wheat *NPF7* genes were responsive to N supply and/or development similarly to the rice *NPF7* orthologues. For example, *TaNPF7.6* is among the most highly expressed genes in group I (Fig. 3) and was influenced by N supply in leaf and grain (Figs 5, 8). *TaNPF7.3* and *TaNPF7.10* were mainly expressed in root and grain, respectively (Fig. 3), and the developmental expression patterns were further verified by RT–qPCR.

Among the eight *NPF* subfamilies of wheat, *NPF5* is the largest subfamily and includes as many as 34 members. Combination of the RNA-seq and RT–qPCR analysis suggests a physiological role for some wheat *NPF5* genes. *TaNPF5.20* was mainly expressed in vegetative tissues, and was up-regulated by N supply in leaves, stems, and nodes, but not in roots. *TaNPF5.26* and *TaNPF5.30* transcripts were concentrated in roots, although at different expression levels, and showed up-regulation by N supply in roots. In Arabidopsis, three tonoplast-localized *NPF5* genes, *AtNPF5.11*, *AtNPF5.12*, and *AtNPF5.16*, have been reported to be involved in vacuolar nitrate efflux and reallocation. *TaNPF5.20*, *TaNPF5.26*, and *TaNPF5.30* may be involved in nitrate reallocation in different tissues and govern the balance of nitrate between the cytoplasm and vacuole in response to the changeable N supply. Dipeptide transport activity has been verified so far only for Arabidopsis subfamily 8 NPF and one member of subfamily 5, AtNPF5.2 (Supplementary Table S12).

Members of the wheat NPF subfamily 8 have been shown to be strongly developmentally regulated in different tissues, and partly regulated also by N availability. For example, gene expression of TaNPF8.4 and TaNPF8.9 is up-regulated post-anthesis in nodes and stems, in contrast to TaNPF8.19, TaNPF8.24, and TaNPF8.28, which are down-regulated. Different expression patterns of wheat subfamily 8 have also been found in other tissues, suggesting a developmental/tissue-specific and N-dependent regulation of dipeptide distribution within the plant.

As already mentioned, Arabidopsis NPFs of nearly all subfamilies are also involved in plant hormone transport (Supplementary Table S12). The different plant hormones have crucial functions in controlling nearly all aspects of plant growth and development. In addition to the role as a nutrient, nitrate acts as a signal, and N nutrition and plant hormone signalling pathways are closely interconnected (Vega *et al.*, 2019). In Arabidopsis, the auxin transport activity of NPF6.3 regulates N auxin accumulation in lateral roots which prevents lateral root elongation and outgrowth (Krouk *et al.*, 2010). Gene expression of wheat NPF orthologues TaNPF6.2 and TaNPF6.3 is regulated by N availability (Buchner *et al.*, 2014). Two potential orthologous auxin-transporting NPFs may provide wheat with a more sensitive modulation of root system architecture in relation to N availability. In addition to the stress response, ABA plays essential roles in different physiological processes. Loss-of-function mutants of the vascular-located AtNPF4.6 exhibit less sensitivity to ABA during seed germination and seedling growth (Kanno *et al.*, 2012). Four out of seven Arabidopsis NPF subfamily 4 have been identified as ABA transporters (Supplementary Table S12). Additional members of the NPF subfamilies 1, 2, 5, and 8 have been confirmed to be able to transport ABA. Long-distance transport of ABA regulates stomatal activity in relation to water availability (Kanno *et al.*, 2012) as well as promoting tiller bud dormancy in cereals (Luo *et al.*, 2019). Short-distance ABA transport has been shown to influence root growth by accumulation of ABA in the root meristem, as well as root hair growth (Ondzighi-Assoume *et al.*, 2016; Rymen *et al.*, 2017). Transcripts of orthologous wheat NPFs are present in all tissues, partly developmentally and/or nitrogen regulated. Involvement in ABA transport may be possible but needs to be verified.

GAs are involved in many developmental processes such as seed germination, root and shoot elongation, flowering, and fruit patterning. In almost all subfamilies, GA-transporting NPFs are present with different affinities for different active GAs (Chiba *et al.*, 2015). Overexpression of AtNPF3.1 enhances GA_3_ flux into all cells of the root, and *npf3* mutants have impaired hypocotyl elongation and seed germination. The post-anthesis developmental up-regulation of the direct wheat orthologue, TaNPF3.1, in the grain, leaves, and stems suggests more participation in N- than GA-related transport involved in N remobilization and N transport to the grain. In contrast to the single Arabidopsis NPF3, there are three further indirect orthologous subfamily 3 NPFs which may be potentially involved in GA transport. The majority of the orthologous Arabidopsis subfamily 2 NPFs are able to transport GA and jasmonate as well as nitrate (Supplementary Table S12), indicating a potential dual action in relation to the substrate (nitrate and/or hormones), and involvement in hormonal control in relation to nutrition as well as development. Mutations of rice OsNPF2.2 and OsNPF2.4 resulted in severe dwarfism and reduced panicle length (Li *et al.*, 2015; Xia *et al.*, 2015), indicating involvement in GA transport. Different wheat subfamily 2 NPFs are developmentally, N only, or developmentally/N regulated in different tissues, which does not exclude possible activity in GA transport.

### Strategies for improvement of crop productivity

Transgenic approaches by overexpression of *NPF* and other nitrate transporter genes have been successfully used to improve crop productivity and NUE in rice (Hu *et al.*, 2015, Chen *et al.*, 2016; Fan *et al.*, 2016; J. Wang *et al.*, 2018), maize (Allen *et al.*, 2016), and tomato (Fu *et al.*, 2015). Overexpression of *OsNPF6.5* (wheat orthologues *TaNPF6.2*, *6.3*), *OsNRT2.1* and *OsNRT2.3b* in rice, and *ZmNRT1.1A* (wheat orthologue *TaNPF6.1*) in maize increased grain yield, above-ground biomass, and NUE. Overexpression of *OsNPF6.3* (wheat orthologue *TaNPF6.1*) in rice significantly shortened maturation time by 9–13 d and 10–18 d under low and high N conditions, respectively, with the grain yield per plant being increased by 32–50% (W. Wang *et al.*, 2018). Tiller number and grain yield were also increased in rice by overexpression of four *OsNPF7* subfamily genes (Supplementary Table S12). The *NPF* gene expression profiles in different wheat tissues in this study indicate additional promising candidates. Further approaches are necessary to understand the specific functions of these candidate genes for optimizing productivity and NUE, by manipulation of these *NPF* genes in future wheat breeding.

### Conclusions

In this study, we performed a systematic analysis of the wheat *NPF* gene family, including gene composition, chromosome locations, and phylogenetic relationships. We carried out detailed RNA-seq and experimental analysis to identify *NPF* gene expression responses in relation to tissue specificity, abiotic and biotic stresses, and more closely to N supply and/or development in different tissues. Our experimental analysis was based on materials derived from field trial experiments for verification of the putative roles and functions of individual *NPF* genes in N utilization. The results offer a foundation for future work aimed at both elucidating the molecular mechanisms underlying *NPF* gene functions in N utilization and optimizing productivity and NUE by manipulation of these *NPF* genes in wheat.

## Supplementary data

Supplementary data are available at *JXB* online.

Fig. S1. RNA-seq expression profiles of selected *NPF* genes.

Fig. S2. Validation of the expression profiles of selected *NPF* genes by RT–qPCR analysis.

Fig. S3. Heatmap of *NPF* expression profiles in relation to different stresses.

Fig. S4. Heatmap of expression profiles of non-regulated *NPF* genes in roots at growth stages Z23 and Z45 by RT–qPCR.

Fig. S5. Heatmap of post-anthesis expression profiles of non-regulated *NPF* genes in nodes by RT–qPCR.

Fig. S6. Heatmap of *NPF* expression profiles in spikes by RT–qPCR.

Table S1. Analysis of Arabidopsis NPF protein domain.

Table S2. Wheat *NPF* gene candidates containing the IPR000109 protein domain.

Table S3. List of wheat genes excluded from the *NPF* gene family.

Table S4. Classification of the *NPF* gene family in wheat.

Table S5. Chromosomal locations of wheat *NPF* genes.

Tables S6. RNA-seq expression data of Choulet *et al.* (2014).

Table S7. Fold change (≥3-fold) of *NPF* gene expression under various abiotic and biotic stresses

Table S8. Comparison of identified wheat *NPF* genes with Bajgain *et al.* (2018).

Table S9. Identification of non-*NPF NRT* genes in Bajgain *et al.* (2018).

Table S10. Primer sequences used for RT–qPCR expression analysis.

Table S11. Normalized relative expression (NRE) data of RT–qPCR expression analysis.

Table S12. Summary of potential functions of identified *NPF* genes in Arabidopsis and rice, and their phylogeny orthologues in wheat.

Protocol S1. Detailed explanation about the *NPF* gene selection for RT–qPCR gene expression analysis

eraa210_suppl_Supplementary-Figures-S1-S6

eraa210_suppl_Supplementary-Tables-S1-S11

eraa210_suppl_Supplementary-Table-S12

eraa210_suppl_Supplemental-Protocol-S1
