# Supplementary material for: Phylogeny and gene expression of the complete NITRATE TRANSPORTER 1/PEPTIDE TRANSPORTER FAMILY in Triticum aestivum
Source: J Exp Bot. 2020 May 28;71(15):4531–46. doi: 10.1093/jxb/eraa210 (PMC7382379; doi:10.1093/jxb/eraa210)
Supplement: eraa210_suppl_Supplementary-Figures-S1-S6 [file eraa210_suppl_supplementary-figures-s1-s6.pdf]

Supplementary Figures S1-S6

Supplementary Figure S1

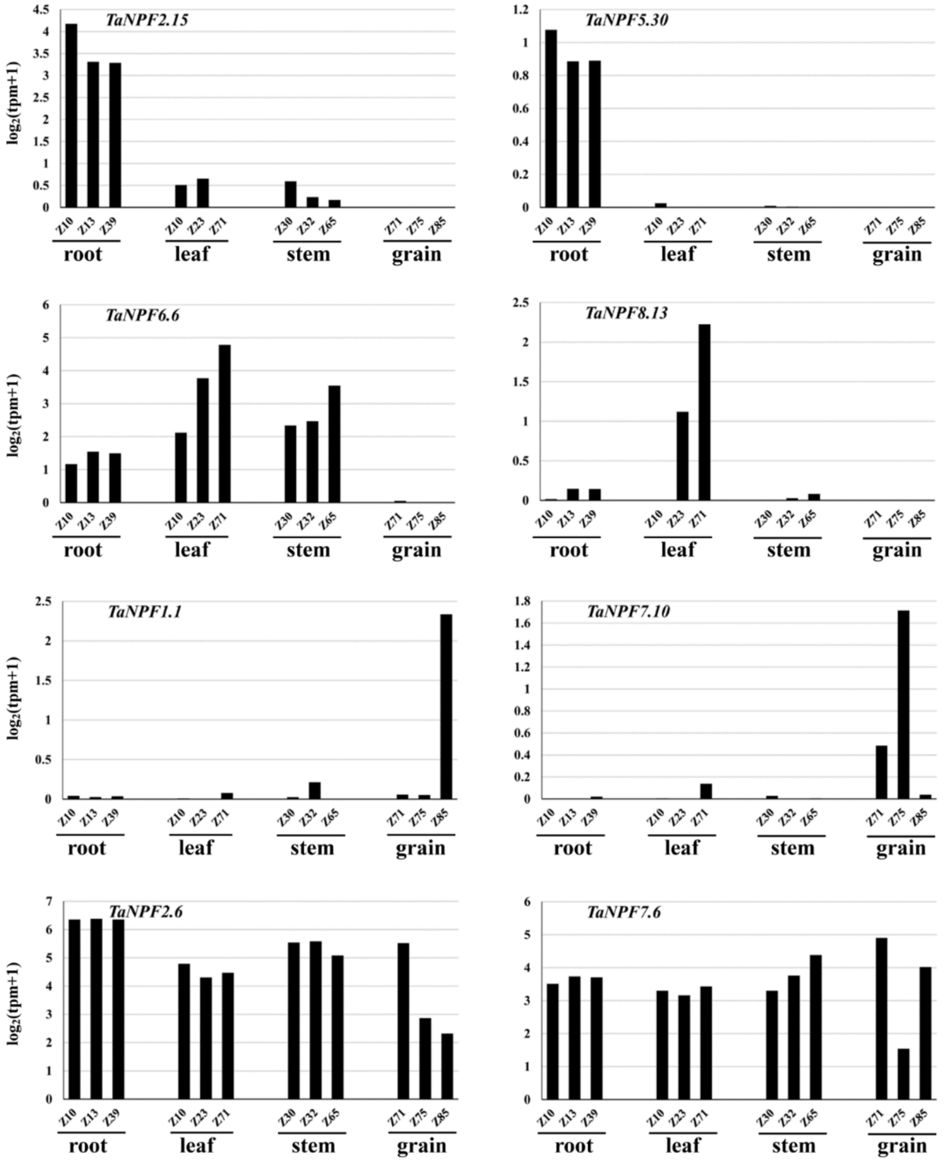

Figure S1. Expression profiles (log$_2$(tpm+1)) of the RNA-seq data in Choulet *et al* (2014) of expression groups selected eight *NPF* genes in different wheat tissues at different growth stages. Z10: seedling stage, Z13: three leaf stage, Z23: tillering stage, Z30: 1 cm spike, Z32: two nodes detectable, Z39: flag leaf stage, Z65: anthesis, Z71: 2 days post anthesis, Z75: 14 days post anthesis, Z85: 30 days post anthesis.

Supplementary Figure S2

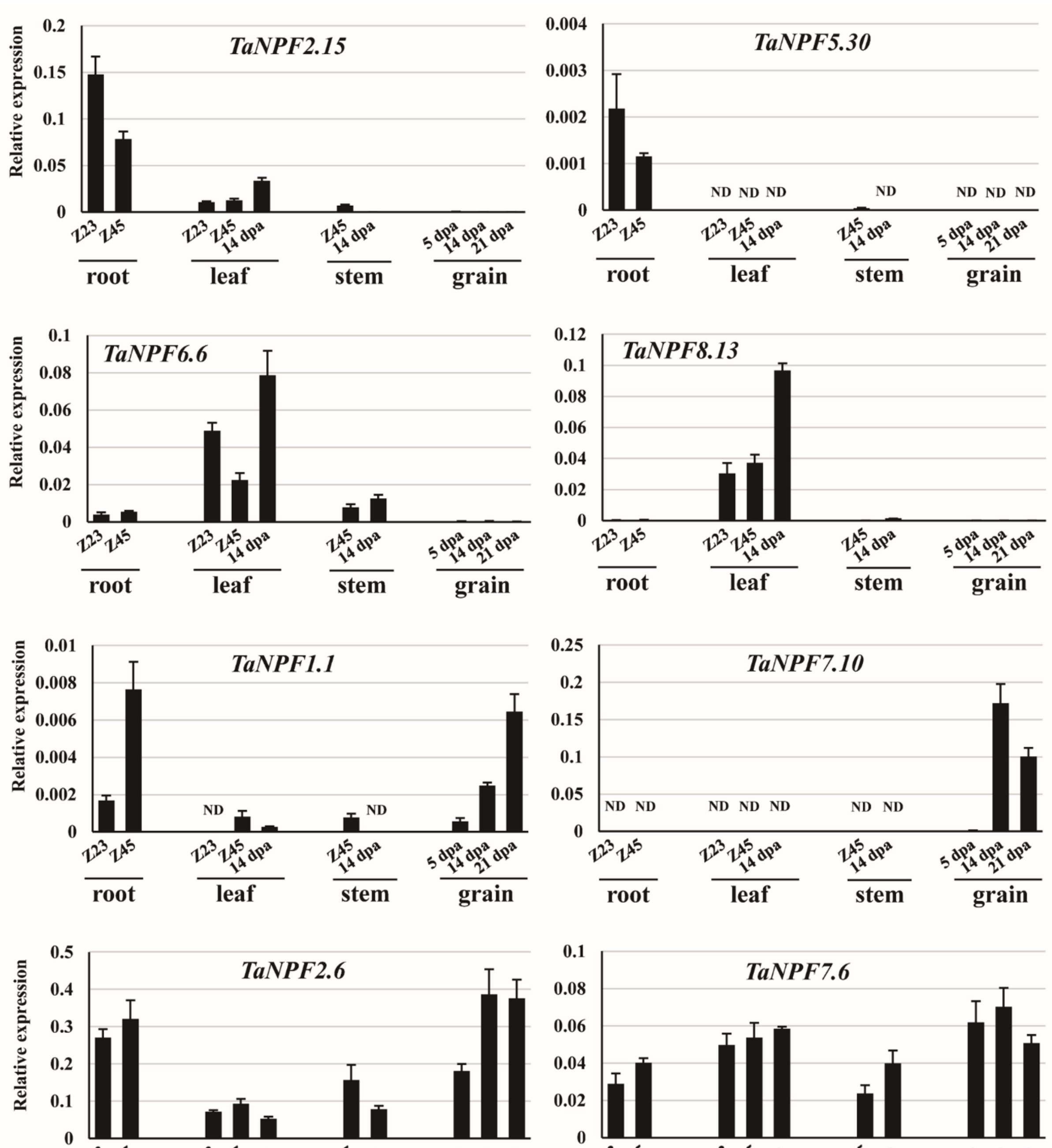

Figure S2. Validation of the expression group profiles of selected *NPF* genes by RT-qPCR analysis. Expression analyses was performed in various wheat tissues at different growth stages (n=3 replicates). Z23: tillering stage, Z45: booting stage, dpa: days post anthesis, ND: not detected.

Supplementary Figure S3

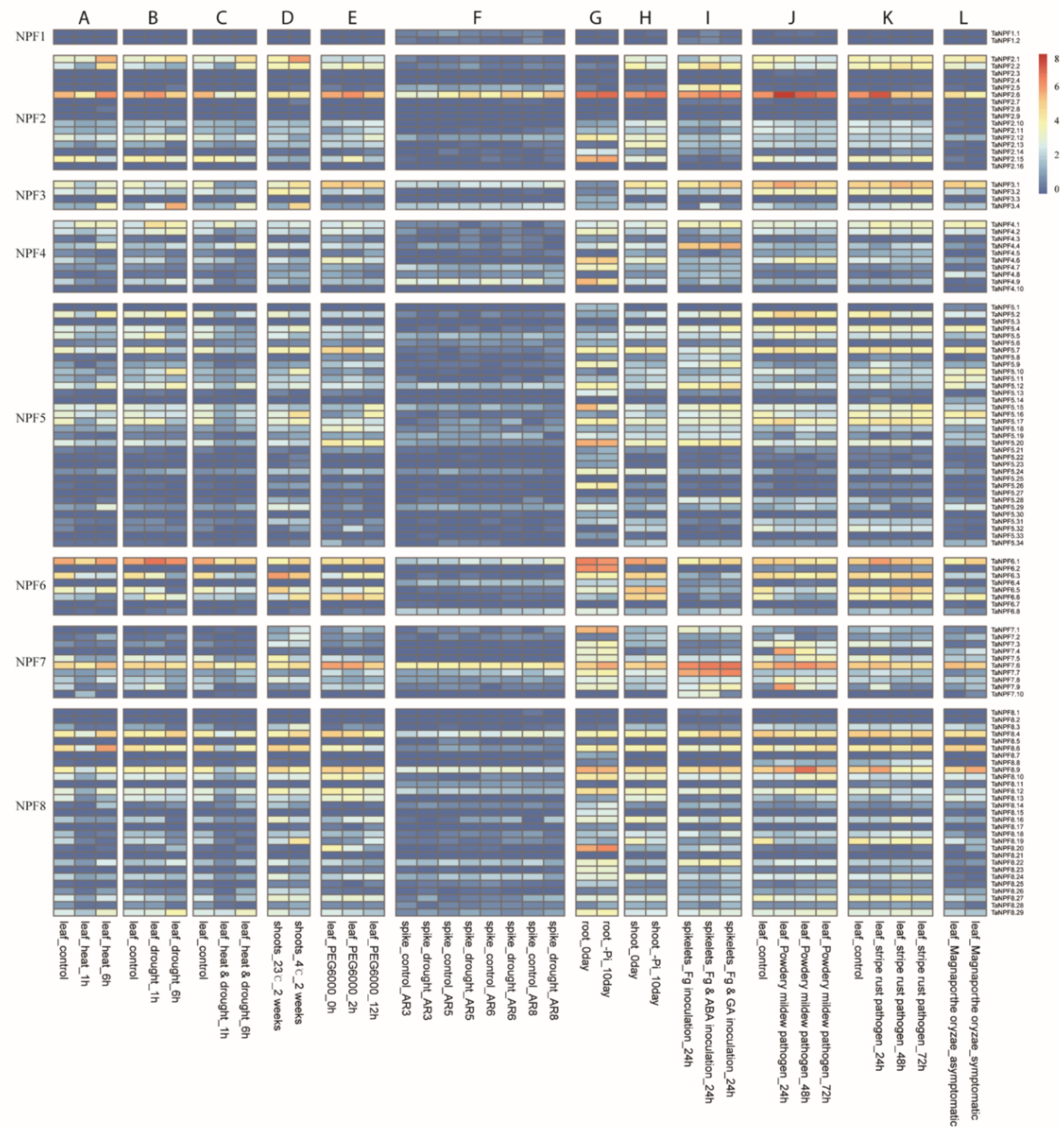

Figure S3. Heatmap of *NPF* expression profiles in relation to abiotic and biotic stresses extracted from exp VIP Wheat expression Browser (Ramírez-González *et al*., 2018). A-C: Heat-, drought-, and drought-heat stress (Liu *et al*., 2015); D: Cold –stress (Li *et al*., 2015); E: PEG stress (N/A); F: Spike drought stress (Ramírez-González *et al*., 2018); G-H: Phosphate starvation stress roots and shoots (Oono *et al*., 2013); I: Spikelets Fusarium/ABA/GA stress (Buhrow *et al*., 2016); J-K: Leaf powdery mildew/stripe rust stress (Zhang *et al*., 2014); L: Leaf fungal *Magnaporthe oryze* stress (Islam *et al*., 2016). Data are based on transformed (log_2(tpm+1)) values of RNA-seq data.

Supplementary Figure S4.

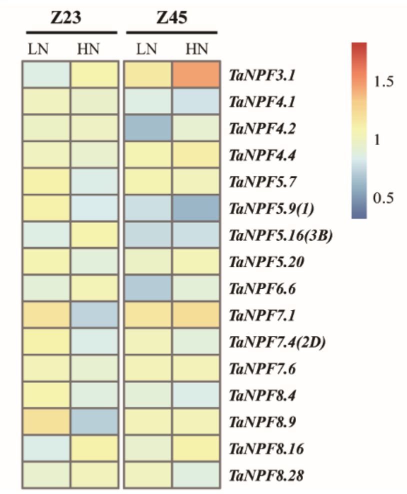

Figure S4. Heatmap of expression profiles of non-regulated *NPF* genes in roots at growth stages Z23 and Z45 by RT-qPCR. Gene expression data were normalized per each gene and shown as log_2-transformed data of normalized data + 1. LN: low nitrogen (0 kg/ha) application, HN: high nitrogen (200 kg/ha) application.

Supplementary Figure S5

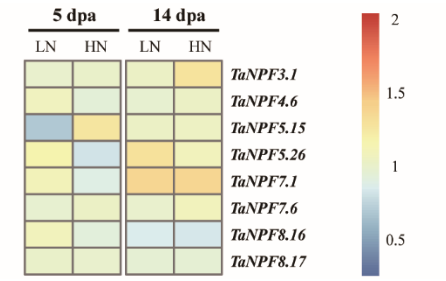

Figure S5. Heatmap of post-anthesis expression profiles of non-regulated *NPF* genes in nodes by RT-qPCR. Gene expression data were normalized per each gene and shown as log2-transformed data of normalized data + 1. dpa: days post anthesis, LN: low nitrogen (0 kg/ha) application, HN: high nitrogen (200 kg/ha) application.

Supplementary Figure S6

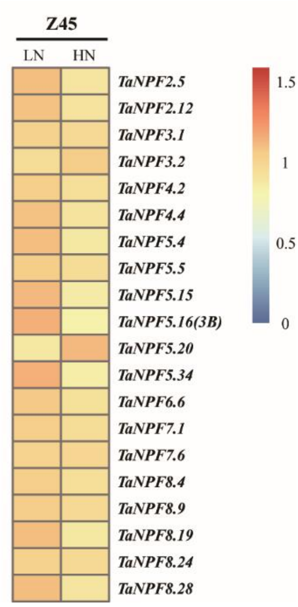

Figure S6. Heatmap of *NPF* expression profiles in Spikes by RT-qPCR. Gene expression data were normalized per each gene and shown as $\log_2$-transformed data of normalized data + 1. LN: low nitrogen (0 kg/ha) application, HN: high nitrogen (200 kg/ha) application.
