# Supplementary material for: Phylogeny and gene expression of the complete NITRATE TRANSPORTER 1/PEPTIDE TRANSPORTER FAMILY in Triticum aestivum
Source: J Exp Bot. 2020 May 28;71(15):4531–46. doi: 10.1093/jxb/eraa210 (PMC7382379; doi:10.1093/jxb/eraa210)
Supplement: eraa210_suppl_Supplemental-Protocol-S1 [file eraa210_suppl_supplemental-protocol-s1.pdf]

Supplemental Protocol S1

Selection of individual *NPF* gene subset for RT-qPCR expression analysis

Candidate wheat *NPF* genes for RT-qPCR analysis were mainly but not strictly selected according to the RNA_seq data (Fig. 3, Choulet *et al*., 2014). The RNA_seq data of wheat NPF family were arranged by summing tpm (transcript per million) values of different developmental stages in respective tissues. Then the genes were ranked by tpm values from high to low in each tissue, respectively. In the first step, candidates among 113 homoeologous groups had to be selected for subsequent RT-qPCR analysis. The table below shows the integrated results ranked by root tpm values. Gray shaded cells/bold fonts indicate the *NPF* gene selected and subjected to RT-qPCR analysis in the corresponding tissue(s) (green shaded). *NPF* genes 1-14 includes members which are relatively high specific expressed in roots and members also expressed in other tissues. 9 other *NPFs* (*TaNPF5.16*, *TaNPF7.9*, *TaNPF5.26*, *TaNPF7.4*, *TaNPF8.15*, *TaNPF2.11*, *TaNPF4.6*, *TaNPF5.30* and *TaNPF8.17*) were further selected for preferring expression in root even though with lower expression levels at different degrees representing a *NPF* gene cluster according to their characters of highly and preferred gene expression in roots. Similarly, *NPF* gene clusters that highly or mainly (specific) expressed in leaf, stem, spike and grain were further selected. Some candidates were repeatedly selected due to their constitutive and relative high expression in all tissues. In summary, 53 non-redundant *NPF* genes were finally selected.

Secondly, RT-qPCR primers were designed for 46 out of the 53 *NPF* genes because primers of the other 7 *NPF* genes had been reported and verified by Buchner and Hawkesford, 2014. RT-qPCR primers for 40 *NPF* genes could be successfully designed and verified but failed for the other 6 *NPF* genes (*TaNPF7.9*, *TaNPF8.6*, *TaNPF5.29*, *TaNPF4.9*, *TaNPF3.4* and *TaNPF6.7*) even though different strategies (degenerate bases, pointing at specific homeolog etc.). Finally, RT-qPCR primers for 47 out of 53 *NPF* genes could be obtained.

In a third step *NPF* genes responses was identified to both, development and nitrogen application in individual tissues. Those *NPF* genes were analysed with high expression in the target tissue(s) but not necessarily in all tissues. As there were no RNA_seq data for node gene expression for reference, we referred mainly (but not limited) to RNA_seq data of stem.

Above are procedures for selection and subsequently detection of *NPF* genes in various wheat samples. We have to admit in following:

1. There were still *NPF* genes that showed preferred expression in individual tissues and were not selected for RT-qPCR analysis as almost all the genes were differentially expressed in different tissues.

2. The RNA_seq data tell us useful information but not all. During the RT-qPCR process, detection of some *NPF* genes were not strictly according to RNA_Seq tpm values in tissues. These modifications were mainly based on the consideration of tissue classification by vegetative and reproductive. For example, *TaNPF2.6* is the most abundantly expressed *NPF* gene in all the tissues but was subjected to RT-qPCR only in the vegetative root and the reproductive grain; *TaNPF5.15* expressed highly in vegetative root and stem, but it was

checked in reproductive spike and grain beside the root. In addition, extending of *NPF* gene detection from its mainly (specific) expressed tissue to other tissue(s) was also due to exploration based on the available primers. For example, *TaNPF6.3*, though expressed higher in root than in leaf, was selected by its high ranking in leaf, and was further checked in stem and node besides in leaf, and the results indicated that *TaNPF6.3* expression pattern in stem and node was very different from that in leaf.

3. There were 6 exceptions that the *NPF* gene expression was not detected based on the original RNA_seq expression data. For example, *TaNPF5.7* was firstly considered to be detected in leaves but it was checked in the secondly considered tissues root and stem. Similar situations were also found in the analysis of the other 5 *NPF* genes (*TaNPF2.11*, *TaNPF4.6*, *TaNPF8.16*, *TaNPF2.10* and *TaNPF3.2*). Although these 6 *NPF* genes were not detected according to original plan as a result from our incomplete consideration, their results of RT-qPCR were reliable.

4. In the RT-qPCR experiment, we totally selected and detected 47 out of 113 *NPF* genes in wheat.

Not all of the results were shown in the text and reasons were listed below:

i) *TaNPF6.1* was actually detected not only in field samples of this text, but also in hydroponic samples and different wheat backgrounds with different nitrogen treatments. We cloned *TaNPF6.1* in wheat before this experiment, and we are now focusing on its detail functions in nitrogen utilization. So, we decided not to show its expression pattern here and to report it in detail together with its physiological function in wheat nitrogen utilization.

ii) *TaNPF6.2* was almost specific expressed in root, and it was indeed selected and checked. But the results revealed that there were large errors among biological replications of high nitrogen samples at Zadoks45 even though repeated. Similar situations were also found in *TaNPF5.9* (in stem), *TaNPF7.7* (in spike), *TaNPF4.6* (in stem), *TaNPF8.16* (in spike), *TaNPF8.19* (in stem) and *TaNPF1.2* (in spike). In other situations, *TaNPF6.4* showed abnormal amplifications in shoot tissues (leaf, stem and spike) when checking amplification curve and annealing temperature, indicated non-specific amplification in shoot tissues but not in root. *TaNPF6.2* and *TaNPF5.9* were also detected in leaf and spike, respectively, but yielded no signal of amplification.

These results mentioned above were not shown in the text. In summary, we selected totally a subset of 47 *NPF* genes, and subsequently identified their expression pattern mainly according to the RNA_seq data. During the process, there was randomness to some extent when considering the modifications of implementation and incomplete consideration mentioned above. After an overall arrangement of the expression data, 28 (in root), 16 (in leaf), 20 (in stem), 27 (in node), 20 (spike) and 16 (in grain) *NPF* genes that integrate into 44 *NPF* genes were finally shown in the text. We have supplemented some explanations in the manuscript text.

	NPF gene	root	leaf	stem	node	spike	grain
1	***TaNPF2.6***	732	200	376	-	361	166
2	***TaNPF5.15***	171	10	130	-	10	23
3*	***TaNPF6.1***	143	69	131	-	106	57
4	***TaNPF2.15***	142	5.4	4.4	-	1.0	0.0
5	***TaNPF4.2***	128	21	43	-	7.4	1.0
6	***TaNPF7.1***	127	5.4	41	-	20	5.6
7	***TaNPF5.20***	117	21	30	-	7.4	11
8*	***TaNPF6.2***	117	0.1	0.1	-	0.2	0.0
9	***TaNPF7.6***	116	88	145	-	167	149
10	***TaNPF8.4***	86	43	60	-	63	52
11	***TaNPF8.9***	84	40	77	-	64	36
12	***TaNPF5.9***	84	7.4	51	-	14	5.4
13	***TaNPF2.12***	80	10	36	-	24	0.5
14	***TaNPF7.3***	67	4.2	0.5	-	0.7	1.7
15	***TaNPF6.3***	66	53	10	-	3.2	0.1
16	***TaNPF5.16***	58	12	12	-	13	17
	TaNPF5.17	47	26	19	-	11	10
17*	***TaNPF7.9***	45	6.6	0.9	-	1.1	1.6
18	***TaNPF7.7***	43	11	44	-	92	86
19	***TaNPF5.7***	38	62	20	-	5.7	7.3
	TaNPF5.24	36	8.6	12	-	5.6	3.3
20	***TaNPF5.26***	36	0.9	1.2	-	0.5	0.7
21*	***TaNPF8.6***	35	30	24	-	21	11
	TaNPF8.29	32	10	7.2	-	12	4.7
	TaNPF8.20	32	3.0	7.4	-	16	1.7
	TaNPF5.12	29	8.9	27	-	37	30
	TaNPF8.12	29	18	16	-	22	8.4
	TaNPF7.8	29	9.1	24	-	13	11
22	***TaNPF4.1***	27	50	15	-	22	22
	TaNPF8.23	25	1.5	4.8	-	2.6	0.1
23*	***TaNPF5.29***	24	13	38	-	22	15
24	***TaNPF7.4***	23	0.6	1.0	-	0.7	0.3
	TaNPF8.14	22	2.8	14	-	5.3	0.9
25*	***TaNPF4.9***	22	6.2	44	-	32	0.7
	TaNPF8.10	21	12	6.4	-	3.3	1.8
	TaNPF4.7	21	4.4	6.3	-	10	2.6
26	***TaNPF8.15***	20	0.2	0.5	-	0.7	0.0
27	***TaNPF4.4***	19	8.1	105	-	22	25
	TaNPF8.22	18	5.6	11	-	20	8.3
	TaNPF5.31	17	8.1	12	-	5.0	0.1
	TaNPF8.27	16	12	21	-	4.9	1.0
	TaNPF6.5	16	26	27	-	2.0	0.6
28	***TaNPF2.11***	15	4.0	3.6	-	2.6	0.0
29	***TaNPF6.4***	13	5.7	30	-	29	1.5
30	***TaNPF4.6***	12	5.1	3.4	-	0.2	0.0
31	***TaNPF8.16***	12	20	88	-	17	0.6
	TaNPF6.8	12	5.5	6.1	-	11	11
	TaNPF7.5	9.1	6.0	5.6	-	2.0	1.7
32	***TaNPF8.24***	9.0	18	39	-	14	2.7
	TaNPF5.19	9.0	12	16	-	16	5.7
33	***TaNPF5.30***	8.4	0.1	0.0	-	0.1	0.0
	TaNPF5.13	8.4	0.3	0.4	-	0.5	0.4
34*	***TaNPF3.4***	8.1	11	7.7	-	66	4.1
35	***TaNPF5.5***	7.9	6.3	5.0	-	14	4.4
	TaNPF3.3	7.5	0.1	0.2	-	0.1	0.4
	TaNPF5.23	7.4	0.3	1.6	-	0.3	0.1
36	***TaNPF2.10***	6.6	4.9	5.9	-	7.3	0.0

	NPF gene	root	leaf	stem	node	spike	grain
	TaNPF5.11	6.6	10	5.9	-	4.4	1.3
37	***TaNPF8.18***	6.4	5.4	15	-	9.5	0.1
	TaNPF2.14	5.9	1.6	9.2	-	4.5	0.2
	TaNPF8.7	5.5	0.3	7.0	-	0.2	0.0
38	***TaNPF5.8***	5.3	1.2	3.3	-	6.2	11
	TaNPF5.10	4.8	2.3	4.5	-	0.7	2.2
39	***TaNPF5.4***	4.5	15	9.3	-	46	11
	TaNPF5.22	4.3	0.3	0.5	-	0.5	0.2
40	***TaNPF8.19***	4.3	21	6.4	-	5.8	4.3
41	***TaNPF8.17***	4.0	1.0	3.0	-	0.8	0.1
	TaNPF7.2	3.9	2.6	30	-	14	4.3
	TaNPF5.1	3.7	0.0	0.3	-	0.0	0.0
	TaNPF2.2	3.5	17	24	-	21	13
	TaNPF4.3	3.5	0.4	0.8	-	1.5	0.9
	TaNPF4.5	3.5	2.1	9.6	-	3.6	0.0
	TaNPF5.33	3.5	0.3	0.3	-	0.1	0.0
	TaNPF5.6	3.4	0.9	1.4	-	1.8	1.0
	TaNPF5.21	3.2	0.6	0.4	-	0.5	1.1
	TaNPF5.2	3.2	5.4	9.2	-	13	6.1
	TaNPF8.25	3.2	0.9	4.9	-	7.3	0.5
	TaNPF2.9	2.5	0.0	0.1	-	0.0	0.0
	TaNPF8.26	2.4	2.1	5.9	-	1.0	0.6
	TaNPF5.3	2.3	0.0	0.0	-	0.0	0.0
	TaNPF5.28	2.3	4.0	9.5	-	2.9	0.1
42	***TaNPF6.6***	2.2	47	17	-	3.1	0.1
43	***TaNPF8.28***	1.8	7.0	27	-	6.4	0.5
	TaNPF5.18	1.8	3.2	3.6	-	2.6	1.4
44	***TaNPF3.2***	1.5	3.9	16	-	1.1	2.1
45	***TaNPF3.1***	1.2	24	15	-	25	3.8
	TaNPF8.3	1.0	12	3.0	-	0.6	0.0
	TaNPF2.13	0.9	12	3.2	-	1.9	1.4
46	***TaNPF2.5***	0.9	0.9	1.3	-	22	30
	TaNPF4.8	0.8	3.3	1.6	-	4.4	1.3
	TaNPF8.8	0.7	0.1	0.7	-	0.1	0.0
	TaNPF2.1	0.6	31	9.6	-	18	35
47	***TaNPF8.13***	0.4	9.6	0.2	-	0.1	0.0
	TaNPF2.3	0.3	0.1	0.0	-	0.0	0.0
48	***TaNPF1.1***	0.2	0.2	0.5	-	3.2	12
	TaNPF8.5	0.2	0.1	0.1	-	1.7	0.4
	TaNPF2.16	0.2	0.0	0.0	-	0.0	0.0
49	***TaNPF5.34***	0.2	28	27	-	9.1	0.6
	TaNPF5.14	0.1	0.0	0.1	-	0.0	0.0
	TaNPF2.4	0.1	0.1	0.2	-	0.1	0.0
	TaNPF5.25	0.1	0.2	0.0	-	0.0	0.0
	TaNPF5.27	0.1	0.0	0.0	-	0.0	0.0
50	***TaNPF7.10***	0.0	0.3	0.1	-	0.1	8.1
	TaNPF8.21	0.0	0.1	0.0	-	0.0	0.0
	TaNPF8.2	0.0	0.0	0.0	-	0.0	0.0
	TaNPF2.8	0.0	0.0	0.0	-	0.0	0.8
51*	***TaNPF6.7***	0.0	0.0	0.1	-	8.0	0.1
52*	***TaNPF1.2***	0.0	0.0	0.0	-	10	0.4
	TaNPF8.1	0.0	0.0	0.0	-	0.2	0.3
	TaNPF8.11	0.0	0.6	0.2	-	0.2	0.2
	TaNPF2.7	0.0	0.9	0.0	-	0.1	0.2
	TaNPF4.10	0.0	0.0	0.0	-	0.0	0.0
53	***TaNPF5.32***	0.0	5.3	0.4	-	0.3	0.0

Note: Numerical values in cells were tpm values of RNA_seq data (Choulet *et al*., 2014). The asterisk indicated These NPF genes were not included in the text. Cells filled with yellow, brown, and grey indicated large errors among biological replications, abnormal amplifications, or no detection of amplification, respectively.
